# Empirical evidence for musical syntax processing? Computer simulations reveal the contribution of auditory short-term memory

**DOI:** 10.3389/fnsys.2014.00094

**Published:** 2014-06-06

**Authors:** Emmanuel Bigand, Charles Delbé, Bénédicte Poulin-Charronnat, Marc Leman, Barbara Tillmann

**Affiliations:** ^1^LEAD, CNRS-UMR 5022, Université de BourgogneDijon, France; ^2^Institut Universitaire de FranceParis, France; ^3^Department of Musicology, IPEM, Ghent UniversityGhent, Belgium; ^4^Lyon Neuroscience Research Center, CNRS-UMR 5292, INSERM-UMR 1028, Université Lyon1Lyon, France

**Keywords:** musical syntax, expectancy, auditory short-term memory, modeling, musical brain

## Abstract

During the last decade, it has been argued that (1) music processing involves syntactic representations similar to those observed in language, and (2) that music and language share similar syntactic-like processes and neural resources. This claim is important for understanding the origin of music and language abilities and, furthermore, it has clinical implications. The Western musical system, however, is rooted in psychoacoustic properties of sound, and this is not the case for linguistic syntax. Accordingly, musical syntax processing could be parsimoniously understood as an emergent property of auditory memory rather than a property of abstract processing similar to linguistic processing. To support this view, we simulated numerous empirical studies that investigated the processing of harmonic structures, using a model based on the accumulation of sensory information in auditory memory. The simulations revealed that most of the musical syntax manipulations used with behavioral and neurophysiological methods as well as with developmental and cross-cultural approaches can be accounted for by the auditory memory model. This led us to question whether current research on musical syntax can really be compared with linguistic processing. Our simulation also raises methodological and theoretical challenges to study musical syntax while disentangling the confounded low-level sensory influences. In order to investigate syntactic abilities in music comparable to language, research should preferentially use musical material with structures that circumvent the tonal effect exerted by psychoacoustic properties of sounds.

## Introduction

Music and language are two sophisticated communicative systems that show numerous similarities. During the last decade, several studies have suggested that cognitive and neural resources governing musical syntax processing may be shared, at least partly, with those involved in the syntactic processing of language (for a review, see Patel, 2008). This shared resource hypothesis challenges a modular approach of music cognition (Peretz and Coltheart, 2003), and has several implications for the understanding of music in human species (Peretz, 2006), including the evolution of linguistic and musical abilities (Pinker, 1997; Patel, 2010). Our present study revisits most of the recent research that has investigated musical syntax processing and its interaction with linguistic processing, and we reanalyzed some other referential developmental and cross-cultural studies in the discussion section. We start by summarizing structural similarities between musical and linguistic syntax. We then turn toward the processes that underlie musical syntax processing and we point out some main differences with language processing. Musical syntax organizes listeners' perceptual feelings of tensions and relaxations through time, while linguistic syntax encodes abstract relationships between concepts (Jackendoff, 2009). The distinction between perceptual and symbolic processes is crucial, we claim, for the comparison of musical and linguistic processing. As will be shown below, the musical syntactic features, which have been studied by current empirical research in the field of tonal music perception, lack the abstractness of linguistic syntax and may be parsimoniously accounted for by an auditory short-term memory (ASTM) model. This finding leads us to claim that musical syntax processing, as studied up to now, tells us more about ASTM than about syntactic-like computations, and that further research should perform more rigorous methodological controls of the musical stimuli used.

### Structural similarities between musical and linguistic syntax

The term “musical syntax” describes a set of rules of a given system that organizes musical events over time. Western tonal music is “syntactic” in the sense that it employs perceptually discrete elements (such as tones or chords), which are combined in hierarchical ways to form musical sequences. A further syntactic feature is that musical elements (tones or chords) have particular syntactic functional-psychological qualities that are dependent on the surrounding musical elements, which are furthermore distinct from standard psychophysical properties of pitch (such as metaphorical attributions of high pitch vs. low pitch, cf. Huron, 2006). Sensitivity to these syntactic functional qualities develops spontaneously in most listeners via musical exposure and does not require any special training (see Bigand and Poulin-Charronnat, 2006, for a review). Lerdahl and Jackendoff's (1983) Generative Theory of Tonal Music (GTTM) provided a formal theory of Western musical syntax processing in which the syntactic-like nature of Western music is expressed by reductive forms. Musical events are organized in a strict hierarchy of relative importance so that each event is perceived in specified relationships to surrounding, more important events. There is no syntactic function such as “subject-verb-object” between musical tones or chords, but every event is either an elaboration or a prolongation (weak or strong) of another event (for a review, see Bigand, 1993; Patel, 2008). The prolongational tree, which is reminiscent to Chomsky's (1965) tree structure, specifies the relationships of musical events in an event hierarchy. The prolongational structure is the closest musical counterpart of linguistic syntax (Jackendoff, 2009).

Lerdahl and Jackendoff (1983) formalized the final state of the cognitive representation of a musical piece in a way that allows some comparisons with language. Understanding sentences requires assigning to each word a specific functional relationship with the other words of the sentence. Similarly, understanding music requires assigning to each event a specific function of elaboration or prolongation in relation to the other events. Accordingly, a key feature for comparing music and language is that these musical functions of elaboration and prolongation are *context-dependent*. A given chord, or even a pair of chords, has different functions depending on the tonal context in which it appears. For example, the chords *G* and *C* could act as stable^1^ dominant and tonic chords in the key of *C* major, where the transition from *G* to *C* induces a strong relaxation. The same two chords would act as less stable chords in the key of *F* major, and even as very tensed chords in the key of *B* major (see Krumhansl et al., 1982). A given musical event may thus lead to entirely different tension-relaxation patterns, depending on the key-context in which it occurs (Bigand, 1993). This high context-dependency of musical function is reminiscent of the context dependency of the syntactic function of words in sentences. This fact is generally taken as a strong argument to emphasize the importance of abstract computations in music. “One strong piece of evidence for a cognitivist view of tonal syntax is that certain psychological properties of musical elements derive from their context and structural relations rather than from their intrinsic physical features” (Patel, 2008, p. 260). However, as will be shown below, the present study leads us to re-evaluate this claim.

### The shared syntactic integration resource hypothesis (SSIRH)

The hypothesis that musical and linguistic syntactic computations are resting on similar processes or resources has emerged during the last decade based on several empirical researches (e.g., Patel, 2003, 2008). Accumulated data from electrophysiological and neuroimaging techniques have suggested potential functional and anatomical overlaps between musical and linguistic syntactic processing (see below). Based on these data, Patel (2003, 2008) proposed the “Shared Syntactic Integration Resource Hypothesis” (SSIRH): music and language share neural resources for processes involved in the integration of events, that is the processing of structural relations between chords or words in working memory to create an integrated mental representation. However, knowledge about music and language systems could be stored in distinct neural networks, which could be selectively damaged. This would account for the double dissociations observed for patients with amusia or aphasia (Peretz, 1990; Peretz et al., 1994, 1997).

Following the SSIRH, musical structure processing would involve integrative processes that are as abstract as those involved in language structure processing. This is a challenging and compelling hypothesis that has theoretical (Pinker, 1997; Patel, 2010) and therapeutic implications (e.g., Racette et al., 2006). It raises, however, several questions. As elaborated by Jackendoff (2009), linguistic syntax contains multiple devices for encoding the dependencies among its constituents, but it is difficult to determine which ones may have counterparts in music. At the same time, the prolongational structures in music encode the relative stability of pitch events in local and global contexts, which differs strongly from conceptual relations that linguistic syntax is dealing with. “Thus both on formal and functional grounds, syntax and prolongational structures have little in common, beyond being headed hierarchies” (Jackendoff, 2009, p. 201). An immediate consequence of this difference is that the so-called “syntactic rules in music” are less constraining than linguistic syntactic rules. Scrambling the order of words in a sentence usually has a strong impact on meaning. By contrast, similar manipulations in music have weaker effects, if any. For example, priming effects are affected in scrambled sentences (Simpson et al., 1989), but they are almost unaffected in scrambled music (Tillmann and Bigand, 2001). The perception of tension and relaxation patterns in long chord sequences is weakly affected by the random reordering of chords (Bigand and Parncutt, 1999), and crude alterations of the syntactic organization of real music have only a weak effect on both musically trained and untrained listeners (Karno and Konecni, 1992; Tillmann and Bigand, 1996, 2001; West-Marvin and Brinkman, 1999; Lalitte and Bigand, 2006). Recent fMRI research also revealed that scrambling sentences leads to considerable changes in neural activation patterns, but that the part of the brain responding to that manipulation for language is not at all activated by music (Rogalsky et al., 2011). In sum, this set of findings suggests that integrative syntactic-like processes might not occur in the same way for music and language.

### Syntactic-like computations vs. auditory short-term memory

Musical rules organize the perceptual effects of sound while conceptual information dominates language processing. Language and music thus are likely to tap into different sets of processes, with sensory-driven processes being entangled with syntactic-like processes in music. This is particularly crucial in the case of Western tonal music, because the syntax of this idiom is deeply rooted in the acoustic properties of sounds and their psychoacoustic effects. As such, Western musical syntax reflects both the constraints of acoustic structure (e.g., octave equivalence, harmonic overtones), general auditory mechanisms (e.g., acoustic dissonance, virtual pitch perception, identifying objects, and separating sound sources) and of compositional strategies (e.g., organization of tones in chords and chords in sequences). The latter are historically and culturally based, and exploit the combined acoustic structures and their related perceptual effects in their most expressive ways (see Rameau, 1722). In Western tonal music, structures determined by nature (physical and physiological) and by culture are thus intimately entwined. As a result, the Western tonal hierarchy reflects the sensory properties of musical sounds and their storage in auditory memory. For example, the two events that have the strongest syntactic importance (the tonic and dominant tones or chords) have also strong overlap in harmonic spectra, which is an acoustic fact. In addition, these events will provide evidence for low supporting tones (i.e., *basse fondamentale*, Rameau, 1722; *virtual pitch*, Schouten, 1940), which are psychoacoustic effects. The acoustic properties of sounds thus provide a necessary basis for tonal syntax. This acoustic foundation is claimed to be “necessary but insufficient” (Lerdahl, 2001) and syntactic representations are supposed to intervene in addition. A critical issue thus remains to evaluate how acoustic information stored in ASTM and syntactic representations learned through mere exposure combine for musical syntax processing and to determine their respective weights. This issue has a long history in the field of psychoacoustic, but it has received relatively rare empirical investigations in consideration of its theoretical importance (see Collins et al., 2014, for a recent approach).

Since the 1970s, psychoacoustic research by Terhardt, Parncutt and colleagues pointed out the richness of acoustic information and the contribution of virtual pitches in musical structure processing (see Parncutt, 1989, for a review). For example, Huron and Parncutt (1993) designed a model of tonality perception that was based on Terhardt's pitch perception model (Terhardt et al., 1982a,b) and that took into account virtual pitch perception. Virtual pitches refer to the fact that, when the frequency components of a complex sound are harmonically related (i.e., the ratio between these different partials is simple, e.g., 880 and 1100 Hz, a ratio of 4:5), pitch sensation at their common (fundamental) frequency can be evoked (in our example, 880/4 = 1100/5 = 220 Hz). Even in the absence of any spectral components at that frequency, the “missing fundamental” at 220 Hz is heard. In addition, the model allows estimating the salience (or probability of noticing) of tone sensations evoked by each musical event in a given context. The authors showed that, by taking into account virtual pitches, pitch saliencies, as well as the influence of ASTM decay, the model could account for participants' ratings in Krumhansl's probe-tone experiments, which have led to the establishment of the “cognitive foundation of musical pitch” (Krumhansl, 1990). In Bigand et al. (1996), the cognitive approach of Lerdahl's (1988) TPST, and the psychoacoustic approach of Parncutt (1989) were compared for short chord sequences. Although some advantages of the cognitive view were reported in predicting the perceived tension of chords, the main observation was that cognitive and psychoacoustic models led to similar predictions. Indeed, correlations between Lerdahl's TPST distances and Parncutt's pitch commonality measures were very strong. However, a major problem of all these studies was that psychoacoustic and cognitive predictions resulted from hand-made models that did not start from the auditory signal.

More than a decade ago, some studies (Leman, 2000; Parncutt and Bregman, 2000) used a computational model of ASTM that deals with the auditory signals. They provided convincing evidence that some early findings, which were claimed to support the cognitive approach of music processing (involving the so-called probe-tone experiments, see Krumhansl, 1990), can receive a more parsimonious, sensory explanation. In Leman (2000), auditory-based representation (i.e., auditory images) were calculated from auditory signals and are accumulated in ASTM. The memory traces were echoic images of pitch periodicities that were extracted from the signal by means of a summed-autocorrelation algorithm producing virtual pitches as well as the salience of perceived pitches, largely similar to the tone sensations experienced by listeners. The model evaluated the goodness of fit of a given tone in a musical context by computing its “tonal contextuality,” that is to say, the overlap of its auditory image with the auditory image created by the previous events that were accumulated in ASTM (a complete description of the model is provided in the section “Simulations”). The obtained simulated ratings were highly correlated with the performance of Krumhansl and Kessler's (1982) participants in the probe-tone task. Tones of strong tonal contextuality in ASTM, precisely corresponded to those reported by Krumhansl and Kessler (1982) as being high in the tonal hierarchy. A cognitive view would thus consider Krumhansl and Kessler's (1982) key profiles as reflecting the abstract knowledge of Western listeners about the Western tonal hierarchy. A more parsimonious approach considers these hierarchies as an emergent property of ASTM for pitch (see also Deutsch, 1984; Butler, 1989, for a similar claim). This interpretation has been also proposed on the basis of earlier models, which do not start from the auditory signal. For example, the Krumhansl and Kessler key profiles were accurately modeled with an ASTM approach by Parncutt (1989, pp. 159–160). This approach was refined in Parncutt (1994, Table 3, p. 161) by considering each of the progressions used by Krumhansl and Kessler (1982), and comparing predictions with data for each progression separately. In these simulations, a model combining ASTM and virtual pitch (perceived pitch-class distributions) performed consistently better than a model based on ASTM only (notated pitch-class distributions or stimulus profiles).

Based on the above findings, we believe that evaluating the importance of ASTM for music perception remains a key issue for the understanding of the links between human abilities for syntax processing in music and language. Therefore, the purpose of the present research was to evaluate how much of the musical syntax processing can be accounted for by an ASTM model^2^, without considering additional syntactic-like computation influences. We addressed this issue with a critical reappraisal of the most influential recent studies designed to find empirical support in favor of the syntactic nature of musical processing.

We carried out a set of simulations with the model proposed by Leman (2000). This model proved to be successful for the simulation of a major study in tonal perception (Krumhansl and Kessler, 1982). The model is freely available as a MATLAB toolbox (Leman et al., 2001, see www.ipem.ugent.be/Toolbox), allows starting the simulations from the auditory signal and has been already used in several studies investigating tonal structure processing (e.g., Koelsch et al., 2007; Marmel et al., 2010).

In the next sections, we first describe the auditory model in more detail. We then assess the ability of this model to account for behavioral and neurophysiological data of studies investigating the processing of chord sequences. We then consider the studies reporting interfering effects between simultaneous musical and linguistic syntactic processing, and we address in the discussion section, the simulation of some developmental data, as well as intercultural comparisons. The overall set of simulation is summarized in Table 1, quoting the main outcomes of studies and simulations.

**Table 1:**
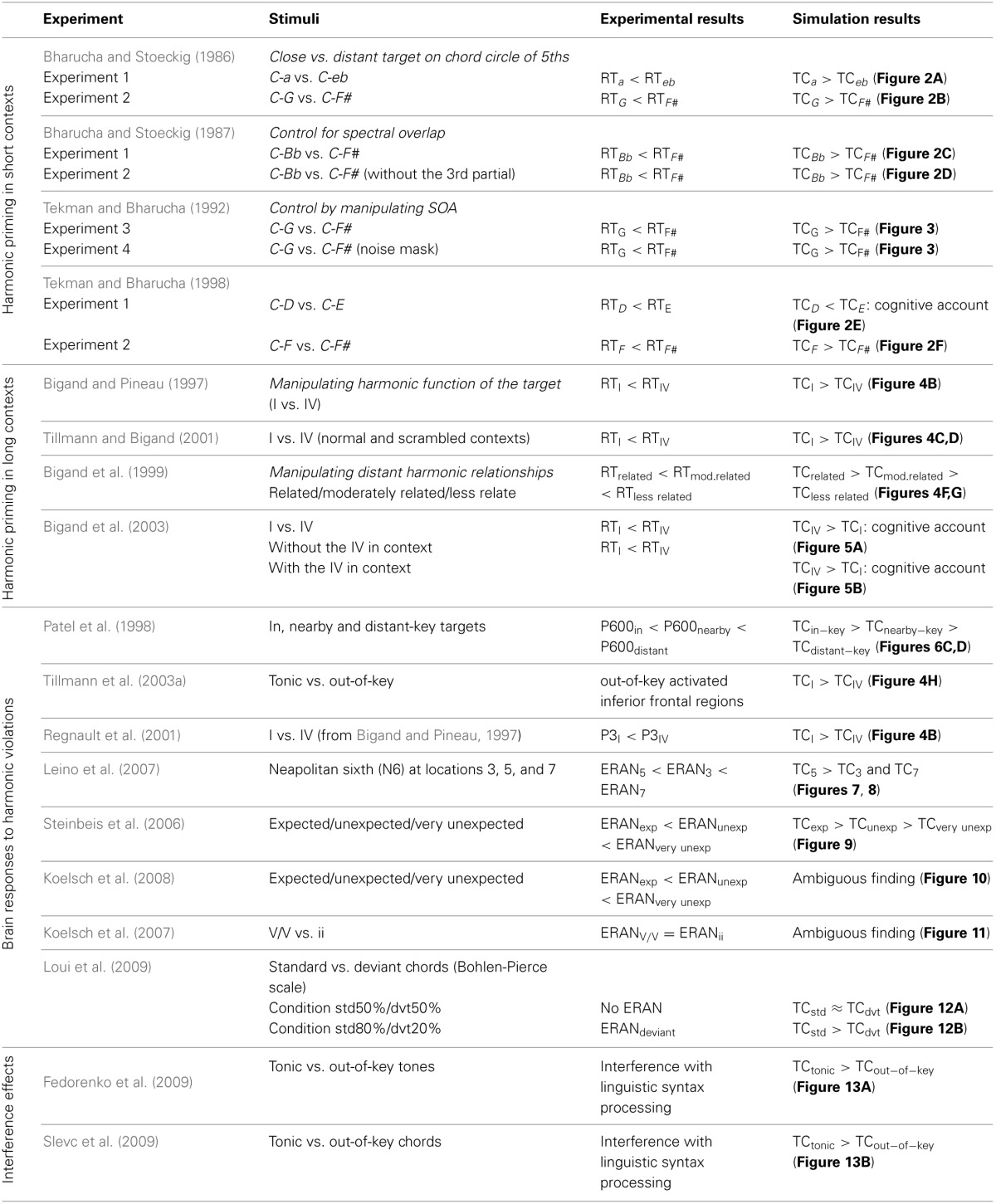
**Summary of experimental studies simulated in the present paper**.

When not otherwise specified, the ASTM model predicted the experimental results. The few cases for which the ASTM model encountered difficulties to account for the data are labeled “cognitive account” or “ambiguous finding.” RT, reaction time; TC, tonal contextuality.

## The auditory short-term memory (ASTM) model

**Figure 1:**
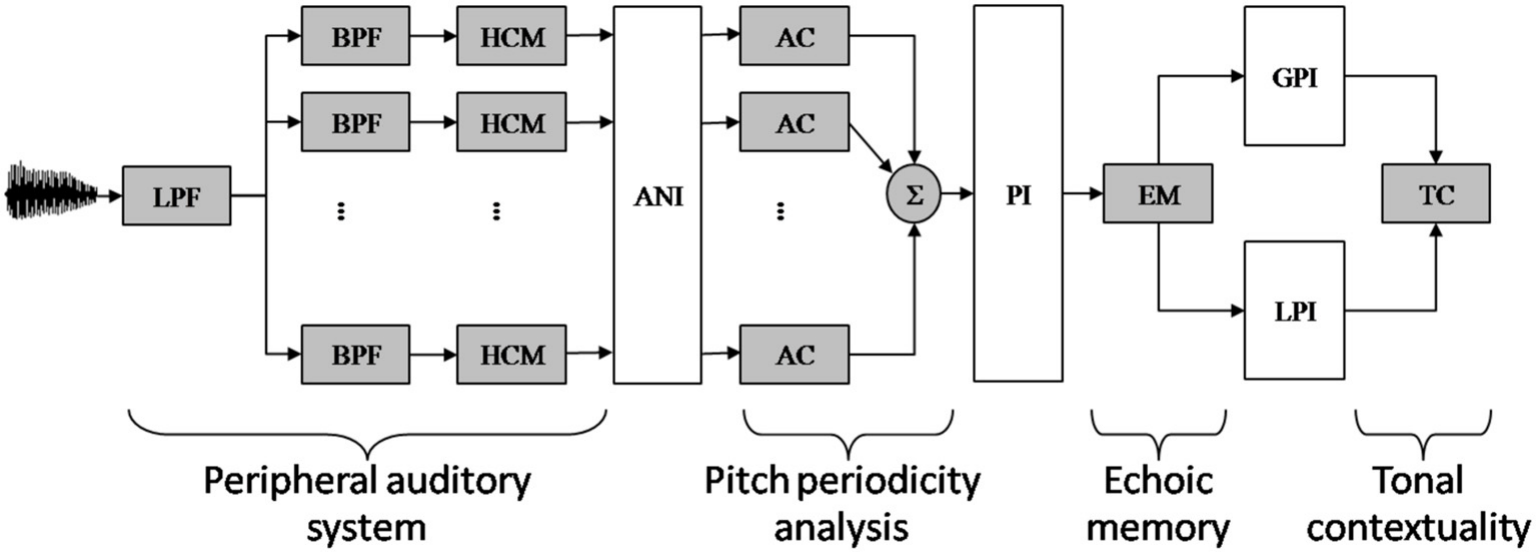
**Schematic diagram of the auditory memory model of Leman (2000)**. The model comprises four components, called peripheral auditory system, pitch periodicity analysis, echoic memory, and tonal contextually. (Component 1) Audio signals are first transformed in neural rate-coded representations by a peripheral auditory model based on Van Immerseel and Martens (1992). The filter characteristics of the outer and middle ear are simulated by a low-pass filter (LPF). The filter characteristic of the inner ear is simulated by an array of 40 band-pass filters (BPF). Half-wave rectification and dynamic range compression simulates the conversion of the band-pass-filtered signals into neural rate-code patterns by the hair-cells (HCM). (Component 2) The resulting auditory nerve image (ANI) is then analyzed by means of a periodicity pitch model, based on a (windowed) autocorrelation (AC) function. The resulting periodicity patterns are then summed up over the auditory channels, resulting in a single summary autocorrelation pattern, called: a pitch image (PI). (Component 3) The echoic memory (EM) model takes a PI as input and gives a leaky integrated PI as output. With very short echo, a Local Pitch Image (LPI) is obtained (i.e., an immediate pitch percept). With a longer echo, taking in consideration more of the preceding contextual information, a Global Pitch Image (GPI) is obtained. (Component 4) The tension of a local pitch image with respect to the global pitch image over time is finally estimated by a similarity measure called the tonal contextuality (TC) index.

The main components of the model are depicted in Figure 1 (for computational details, see Leman, 2000; Leman et al., 2001).

This model comprises four components, called (1) peripheral auditory system, (2) pitch periodicity analysis, (3) echoic memory, and (4) tonal contexuality.

(Component 1). The peripheral auditory system transforms an audio signal into neuronal firing probabilities that simulate patterns of neural firing rate-codes in the auditory nerves. The mathematical details of the signal processing approach that forms the basis of this modeling stage can be found in Van Immerseel and Martens (1992). To illustrate the behavior of the auditory model consider the following example. If two input frequencies are more than a critical band apart from each other, such as 770 and 1100 Hz, they will be resolved separately by a proper processing channel. Accordingly, the neuronal firing probabilities will reflect the phase locking of the neurons to the temporal aspects of the frequencies (the upper range is 1250 Hz). However, if two input frequencies are less than a critical band apart from each other, such as 990 and 1100 Hz, then the neural firing probabilities will not be resolved by separate channels. Instead, the captured frequencies will interfere and the resulting neuronal firing probability pattern will typically reflect an amplitude modulated pattern (on top of a fine temporal pattern reflecting the interfering frequencies that function as a carrier for this amplitude modulated pattern) having a frequency of 110 Hz. Note that this frequency can contribute to a possible virtual pitch at 110 Hz.

(Component 2). The pitch periodicity analysis and subsequent pitch image processing rationale is a slightly adapted version of Van Immerseel and Martens (1992) and has been described in detail in Leman (2000). This component takes neuronal firing probabilities as input and generates summary autocorrelation patterns as output. The processing details of the pitch processing can be checked in the MATLAB routines of the Toolbox (www.ipem.ugent.be/Toolbox). A tutorial has been proposed by Leman et al. (2001). To illustrate the behavior of the pitch periodicity analysis, let us consider the example of a stimulus with frequency components of 600, 800, and 1000 Hz (see Van Noorden, 1982). These frequencies are processed by three different auditory channels of the peripheral auditory system. The channel corresponding to 600 Hz will show periods at multiples of 1/600 (e.g., 1.66, 3.33, 5, 6.66, 8.33 ms), the channel corresponding to 800 Hz will show periods at multiples of 1/800 (1.25, 2.50, 3.75, 5, 6.25 ms), and the channel at 1000 Hz will show multiples of 1/1000 (1, 2, 3, 4, 5 ms). It is easy to see that the period of 5 ms (an integer multiple of the components' periods) is present in all channels; it corresponds to the heard low (virtual) pitch of 200 Hz.

(Component 3). The part called echoic memory does the pitch integration over time. This part takes the summary autocorrelation pattern (i.e., the pitch periodicity analysis pattern) as input and generates two pitch images, just by applying a leaky-integration over time (which is the same as low-pass filtering over time), so that the images become “smeared” over time. One image (called *local* pitch image, or LPI in Figure 1) is obtained by applying a short integration time (e.g., 0.1 s) and it represents the pattern for the immediate pitch percept (i.e., the newly perceived pitch). Another image (called *global* pitch image, or GPI in Figure 1) is obtained by applying a longer integration time (e.g. 1.5 s) and it represents the pattern for the global pitch percept (i.e., the pitch context).

(Component 4). The “tonal contextuality” calculates the similarity between the local image and the global image, using a simple correlation calculation. As we process audio files, we obtain continuous stream of tonal contextuality values, one value at any given time point *t*. These values reflect the degree in which a pitch resembles a pitch context.

For simulation purposes the model is defined by only two parameters, namely, the decay constants that define the integration time for the local and global images (Leman, 2000). Given the importance of those parameters for the simulations described below, we give here a more detailed description of how the ASTM (also called echoic memory, see Figure 1) works.

A leaky-integrated pitch image $\vec{p}$(*n*) is updated at each time step *n* by adding a certain amount of the previous image $\vec{p}$(*n* − 1) using the following equation:

(1) $$\vec{p}\left(n\right)=\vec{p\_in}\left(n\right)+\vec{p}\left(n-1\right)*2^{\frac{-1}{sa*T}},\;n=1,\;2,\;3,\ldots$$

where $\vec{p}$(*n*) stands for the (to be updated) pitch image at a discrete time index value *n*. The input coming from Component 3 is given by $\vec{p\_in}$(*n*). The sample rate is given by *sa*. The half decay time expressed in seconds is given by *T*. Note that the pitch images are represented as vectors. This vector can be interpreted as an array of values where each value represents an evidence for a pitch, similar to the summary autocorrelation pattern described in Component 2. The position in the array can be expressed in periods (or frequency) because this array comes from the autocorrelation analysis of neural firing probabilities that ultimately reflect the periodicities of the frequencies in the audio.

The specified echo *T* defines the amount of context (i.e., expressed as a duration of past pitch images), in seconds, that is taken into account. *T* is a half-decay time. Assume that an impulse is given as input, then $\vec{p\_in}$ will be 1 at the starting position and 0 later on. That is, $\vec{p\_in}$(1) = 1 and for *n* > 1, $\vec{p\_in}$ = 0. Then this value will decay to half its amplitude after *T* seconds. Assume that T is 1 s and that the sample rate is 100 samples per second, then 1 s is reached at *n* = 101. At $\vec{p}$(1) the values of the array will be equal to the input, which is 1. At $\vec{p}$(2) the values will be 1 ∗ $2^{\frac{-1}{100}}$. At $\vec{p}$(2) we start from this value and multiply it by the same amount so that the resulting value will be $2^{\frac{-1}{100}}*2^{\frac{-1}{100}}=2^{\frac{-2}{100}}$. In general the value at *n* time steps we will have the value of $2^{\frac{-(n-1)}{100}}$. Accordingly, after 101 time steps (which amounts to *T* = 100 timesteps = 1 s) the value will be $2^{\frac{-100}{100}}$ = 2^−1^ = 0.5. This shows that the original impulse (value = 1) in the input is reduced to half its value after 100 steps.

The duration that incoming images are kept in short-term memory is defined by the half-decay term *T*. Accordingly, this half-decay term for local and global pitch images influences the values of the tonal contextuality. In Leman (2000), these decays terms were set to 0.1 and 1.5 s, for a local decay and a global decay, respectively, because these values gave the best account of the probe-tone data of Krumhansl and Kessler (1982). In the absence of more precise information about the dynamics of ASTM, these decay parameters are thus free parameters of the model. One new aspect of the present set of simulations is that we systematically manipulate both decay parameters in order to evaluate how these manipulations modulate the outcome of the simulations.

The similarity measure used to estimate the match between the local and global pitch images is referred to as the “tonal contextuality” or “tonal contextuality index” *TC*(*t*). This index reflects the degree of similarity between local and global images at time *t*. Higher *TC* values reflect higher similarity between the two images. The index can be conceived as a representative of the “tension” of a local pitch image with respect to the global pitch image [i.e., high *TC*(*t*) reflecting low levels of tension]. Pearson's correlation coefficient is used for this similarity measure, which was calculated for each experimental item in the different studies. In the present simulations, Fisher's z-transformation was used to convert Pearson's *r* to the normally distributed variable z.

In the simulations reported below, input stimuli to the model were sound files digitized at the standard CD quality (44.1 kHz, 16 bits resolution), which corresponds to the most frequently used quality in music perception research. The mean TC of a given target event in a musical context (*TC*_*target*_), was defined as the mean value of *TC*(*t*) during the time-window of this event. Simulations were carried out with local and global decay parameters varying systematically from 0.1 to 2.5 s by a step of 0.1, in order to explore the complete parameter space of the model for a given stimulus. When appropriate, this complete space of solutions is reported. Positive, negative, and nonsignificant differences between the *TC* of the target chords under consideration are represented by hot, cold, and white colors, respectively (two-tailed paired *t*-test, *p* < 0.05) and *t*-values are reported as solid lines in the figures. The simulations were performed for all the stimuli used in the experiments, and the statistics compared the *TC* values of the stimuli from the syntactically related and unrelated conditions.

## Simulations of context dependency of musical event

### Simulations of local context dependency

Much of the behavioral evidence for syntactic-like processing of Western harmony comes from studies using the harmonic priming paradigm. Bharucha and colleagues investigated priming effects elicited by a single-prime chord (Bharucha and Stoeckig, 1986, 1987; Tekman and Bharucha, 1992, 1998). Participants heard a prime chord followed by a target chord. The prime and target were either harmonically closely related or distantly related. The harmonic relatedness of the two chords was defined on the basis of their music theoretical relationship: the two harmonically related chords shared parent keys, whereas the two harmonically unrelated chords did not. For half of the trials, the pitch of one of the component of the target chord was slightly changed, and participants had to decide as quickly and as accurately as possible if the target chord was in-tune or out-of-tune. Priming effects were revealed by a tendency to judge targets as consonant when they were related to the prime, and by faster reaction times for the related consonant targets than for the unrelated ones. The main problem of the pioneer priming study was that harmonically related chords (such as *C* and *G* major chords) have more component tones in common than do harmonically unrelated chords (*C* and *F*# major chords). Several studies were thus designed to remove this confound. Bharucha and Stoeckig (1987) used related chords that did not have overlap between the harmonic spectra and Tekman and Bharucha (1992) varied the stimulus onset asynchrony (SOA) between prime and target chords aiming to reduce the contribution of ASTM. An elegant study by Tekman and Bharucha (1998) investigated the respective influences of sensory and cognitive priming by using two types of target chords. One target was related to the prime at the sensory level (e.g., *C* and *E* major chords share one component tone), but not at a syntactical level (*C* and *E* major chords do not share any major keys). The other target was unrelated to the prime in terms of pitch information (*C* and *D* major chords share no component tones), but was syntactically related to the prime (both chords belong to the key of *G* major). Facilitated processing for sensory related targets was observed after the short SOA (50 ms), and facilitation for syntactically related targets was observed after the SOAs equal to or longer than 500 ms. This finding suggested that sensory and cognitive priming occur over different time scales: sensory priming dominates over cognitive priming at short delay, but a reverse phenomenon is found for longer delays (i.e., ≥ 500 ms).

**Figure 2:**
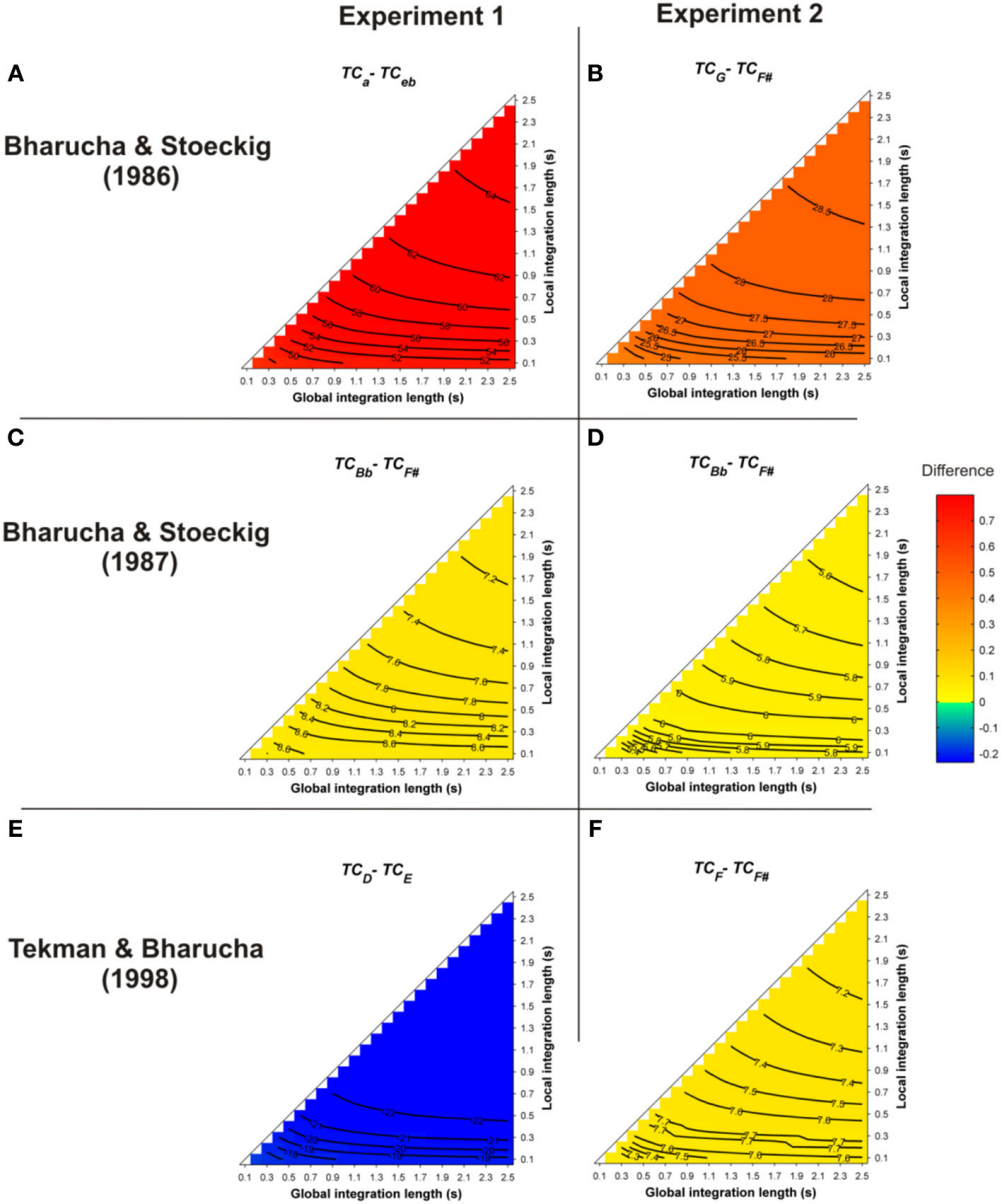
**Mean differences between the *TC* of the target chords (*TC*_*related*_ − *TC*_*unrelated*_), following a single-prime chord, as a function of the local and global context integration windows**. Rows indicate different studies (Panels **A,B**: Bharucha and Stoeckig, 1986; Panels **C,D**: Bharucha and Stoeckig, 1987; Panels **E,F**: Tekman and Bharucha, 1998). Positive, negative, and nonsignificant differences are represented by hot, cold, and white colors, respectively (two-tailed paired *t*-test, *p* < 0.05; *t*-values are reported as contours). A positive difference indicates that the *TC* in ASTM induced by the related target is stronger than that of the unrelated one, thus predicting the harmonic priming effects reported in the behavioral studies. For five out of six experiments, the ASTM model predicted the observed facilitation for the related target over the unrelated target, as reflected by hot colored areas. The targets chords in each experiment are indicated by the indices (lowercase and uppercase letters represent minor and major chords, respectively).

All of these priming data were revisited here with Leman's ASTM model (but see also Parncutt (1989; p. 13) for different behavioral data sets on musical structure processing). The simulations used the stimuli of the behavioral experiments. One hundred and forty-four pairs of chords played with Shepard tones, synthesized as described in Bharucha and Stoeckig (1986), were presented to the model. The prime and target chords sounded for 3 s and 2 s, respectively. The mean differences between the *TC* of the target chords obtained for all of these parameters, i.e., *TC*_*related*_ − *TC*_*unrelated*_, are reported in Figure 2. Warm colors represent a higher *TC* for the harmonically related targets, compared to the unrelated targets. Statistics were computed by comparing the 144 *TC* values of the related and unrelated targets.

**Figure 3:**
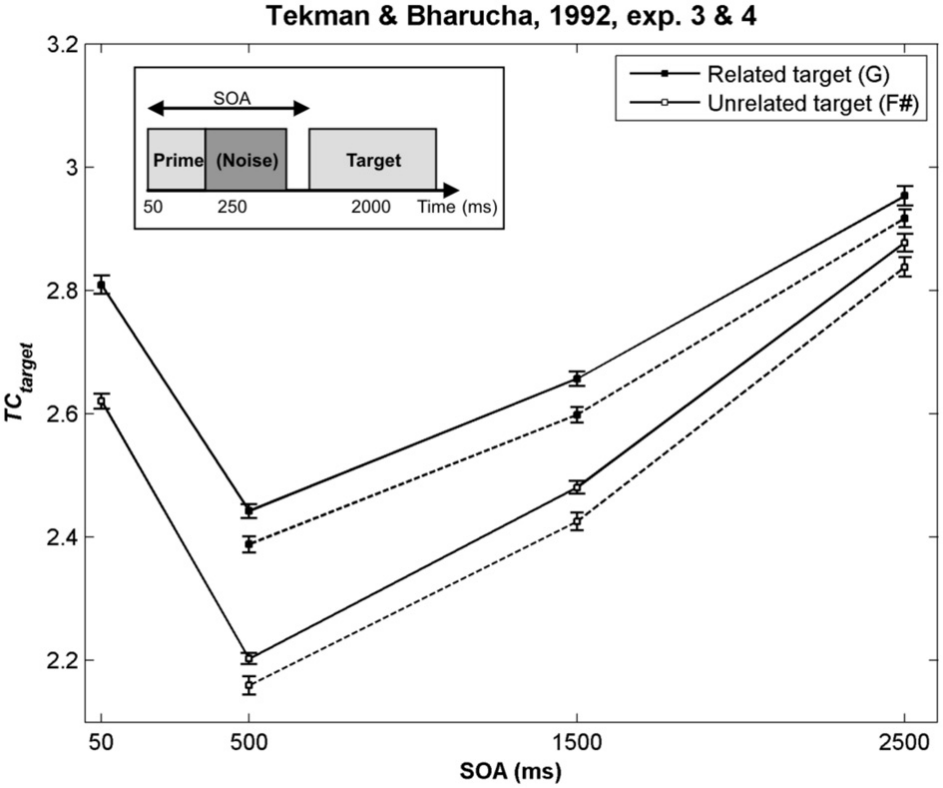
***TC* of the target chords used by Tekman and Bharucha (1992) as a function of the SOA**. In this study, a silence (Experiment 3, solid lines) and a noise (Experiment 4, dash lines) were inserted between the two chords. The prime chord sounded for 50 ms, and could be followed by a silence or a noise of variable duration. The target chord then sounded for 2000 ms (see *insert*). In all cases, the *TC* of the related target chord was higher than that of the unrelated target, thus predicting the facilitation effect observed behaviorally. Error bars represent standard errors between experimental sequences.

As illustrated in Figure 2 by the hot colors, target chords harmonically related to the prime chords have a higher *TC* than unrelated targets (i.e., *TC*_*related*_ − *TC*_*unrelated*_ > 0). This difference predicts facilitated processing for the related targets. This prediction fits with the behavioral data observed by Bharucha and Stoeckig (1986) and Tekman and Bharucha (1998) (Experiment 2). When the *Bb* chord was used as the related target (Bharucha and Stoeckig, 1987, Experiment 1) or when the third partial of the target was eliminated (Experiment 2), the related target was still more strongly primed by the prime chord in terms of *TC*, compared to the unrelated target (Figures 2C,D). Furthermore, whatever the SOA between the chords, a higher *TC* was observed for the related target (*G*; Figure 3, dark circle) than for the unrelated target (*F*#, white circle), and the insertion of silence or white noise (250 ms-length) between the two chords did not change this difference (Tekman and Bharucha, 1992; Figure 3, compare solid and dash lines).

For these eight experiments conducted by Bharucha and Stoeckig (1986, 1987) and Tekman and Bharucha (1992, 1998), the simulations revealed that controlling for the number of tones and spectral tone components shared by prime and target chords was not sufficient to avoid a potential ASTM-based overlap between these chords. Furthermore, the simulations suggest that the manipulation of the SOA and the insertion of a white noise were not strong enough to disrupt ASTM (Figure 3). However, the ASTM model encountered difficulties to simulate Tekman and Bharucha's (1998) data of Experiment 1. The model predicts a stronger *TC* for the harmonically distant, but acoustically related target (*E*; Figure 2E). While this pattern fits with behavioral data at short SOA, it cannot account for the inversion in priming occurring at longer SOAs (see Figure 3). The data reported by Tekman and Bharucha (1998, Experiment 1) are one of the rare empirical data not accounted for by the ASTM model. It might be argued that it is difficult to take this failure of the ASTM model as a convincing demonstration of syntactic-like processing in music. It may simply reflect an incompleteness of the used ASTM model, a point on which we come back in the general discussion.

### Simulations of long context dependency

**Figure 4:**
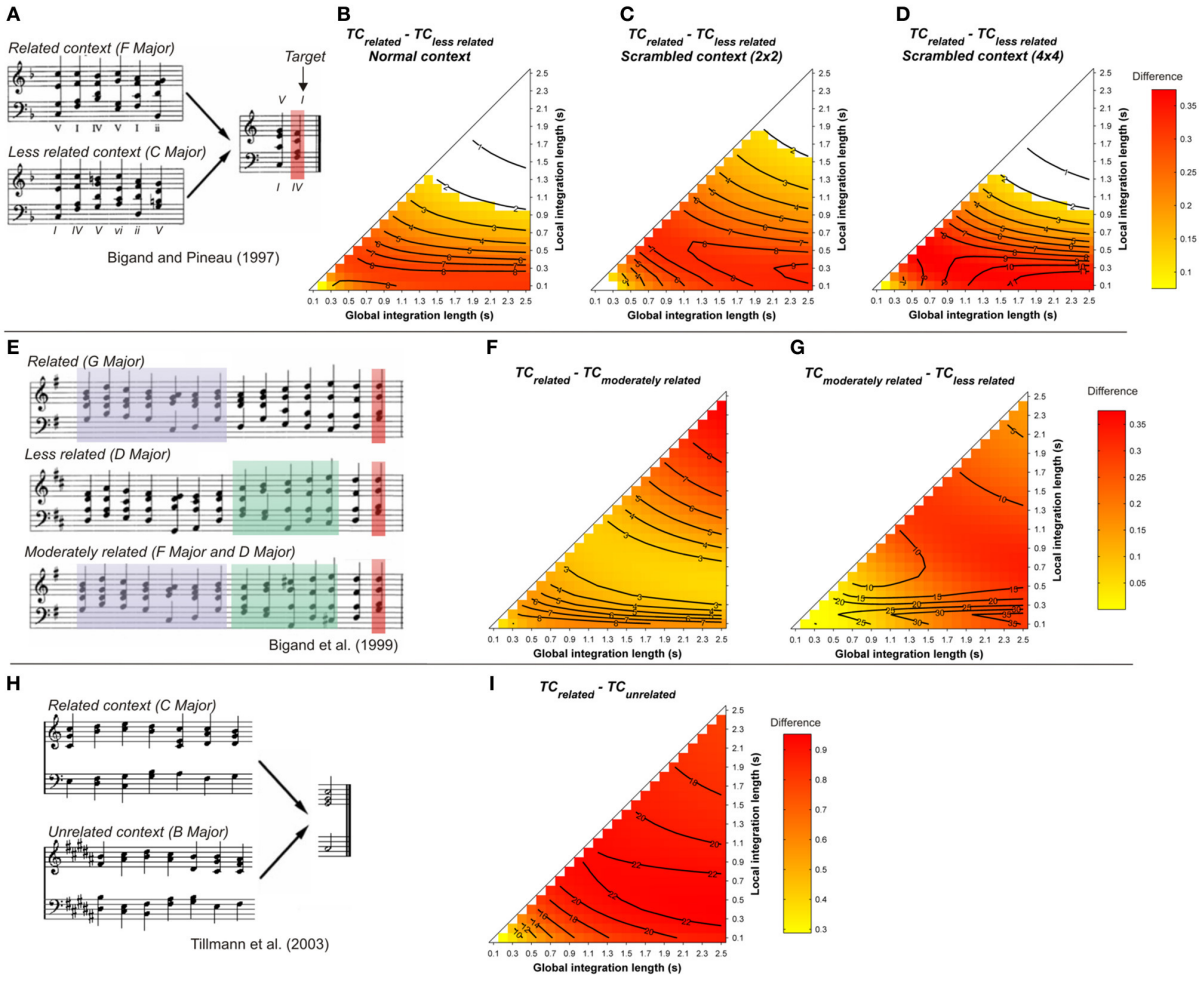
**(A)** Example sequences from Bigand and Pineau (1997). Mean differences between *TC*_*related*_ and *TC*_*less related*_, as a function of the local and global context integration windows, for normal **(B)**, 2 by 2 **(C)**, and 4 by 4 **(D)** scrambled contexts (Tillmann and Bigand, 2001). **(E)** example sequences from Bigand et al. (1999). Mean differences of the *TC* of the target chord between related vs. moderately related **(F)**, and moderately related vs. less related **(G)** contexts. **(H)** example sequences used in the fMRI study of Tillmann et al. (2003a). **(I)** mean differences of the *TC* of the target chord between related vs. unrelated contexts. Positive, negative, and nonsignificant differences are represented by hot, cold, and white colors, respectively (two-tailed paired *t*-test, *p* < 0.05; *t*-values are reported as contours).

In order to assess the effect of long context dependency, Bigand and Pineau (1997) and Pineau and Bigand (1997) created eight-chord sequences in which the two final chords were identical for each pair of sequences. The first six chords (global context) established two different harmonic contexts and changed the harmonic function of the target: a related context, in which the final chord was highly expected (a tonic chord) and a less related one, in which the final chord was less expected (a subdominant chord), while remaining in the key of the context (see Figure 4A). Target chords were processed faster and more accurately in the highly expected condition than in the less-expected condition, indicating an effect of global harmonic context. These chord sequences were used in other studies with behavioral (Bigand et al., 2005, Experiment 3; Tillmann and Bigand, 2001) and electrophysiological measurements (Regnault et al., 2001). Sequences of the harmonically correct (tonic) condition were also compared to their out-of-key-target counterparts in an fMRI study (Tillmann et al., 2003a; Figure 4H). Several brain regions were activated more strongly for the out-of-key targets, notably the inferior frontal regions. Tillmann and Bigand (2001) investigated the effect of scrambling on harmonic priming effects in these chord sequences. They found that, in contrast to sentence priming effects (e.g., Simpson et al., 1989), the temporal organization of the chords in the context did not affect the strength of harmonic priming. In Bigand et al. (1999, Experiment 2), the global context was manipulated at three levels, while holding constant the local context. Harmonic expectancies were thus embedded at two hierarchical levels. The function of the target chord was changed by transposing the preceding chords (except the penultimate one, see Figure 4E). The results provided evidence that musical expectations are dependent on various levels of hierarchical structure. The respective contributions of cognitive and ASTM-based processes was directly investigated in Bigand et al. (2003) by manipulating the occurrence of the target in the harmonic context. In one condition, neither the related (tonic) nor the unrelated (subdominant) target chord occurred in the previous context. In the second condition, the unrelated target was occurring once or twice in the prime context, but not the related target. Hence, in this second condition, the less-expected chord shared many spectral components with the prime context, leading to opposite predictions for ASTM-based and knowledge-driven priming. In this study, behavioral data showed that knowledge-driven priming prevailed over ASTM-based priming in most but not all cases. Consistent with Tekman and Bharucha (1998), ASTM-based priming tended to overrule cognitive priming at extremely fast tempo (75 ms per chord).

Simulations were carried out for all stimuli used in these studies. Original MIDI files from the published studies were used to generate sound files with a piano timbre. In all simulations, chord duration was 600 ms, and the target chord sounded for 1200 ms. Figure 4 reports the mean differences between the *TC* of the related and less-related target chords as a function of the memory parameters. Statistics were performed on the *TC* values of all target chords from the related and unrelated conditions.

The ASTM model accounts for the data on the harmonic priming effects of Bigand and Pineau (1997) and Pineau and Bigand (1997; Figure 4B). The model also accounts for the data of the priming effects reported by Tillmann and Bigand (2001) with scrambled chord sequences (Figures 4C,D). For all these cases, the *TC* of the syntactically related target was higher than the *TC* of the unrelated target, independently of the integration windows, favoring a sensory account of the data. A similar conclusion can be made when the syntactically related-target sequences were compared to their out-of-key-target counterparts, as in the Tillmann et al.'s (2003a) fMRI study (Figure 4I).

The model also replicates the priming effects reported at three hierarchical levels of the musical structure (Bigand et al., 1999). As shown in Figures 4F,G, the differences for both “related *minus* moderately related” and “moderately related *minus* less related” comparisons were positive, as indicated by hot colors. This difference indicates that the similarity of the target chord relative to the harmonic context was always smaller for the unrelated target than for the related target and that, for a given pair of parameters, the *TC* of the target increased with its relatedness with the context (i.e., *TC*_less rel._ < *TC*_moderately rel._ < *TC*_rel._).

**Figure 5:**
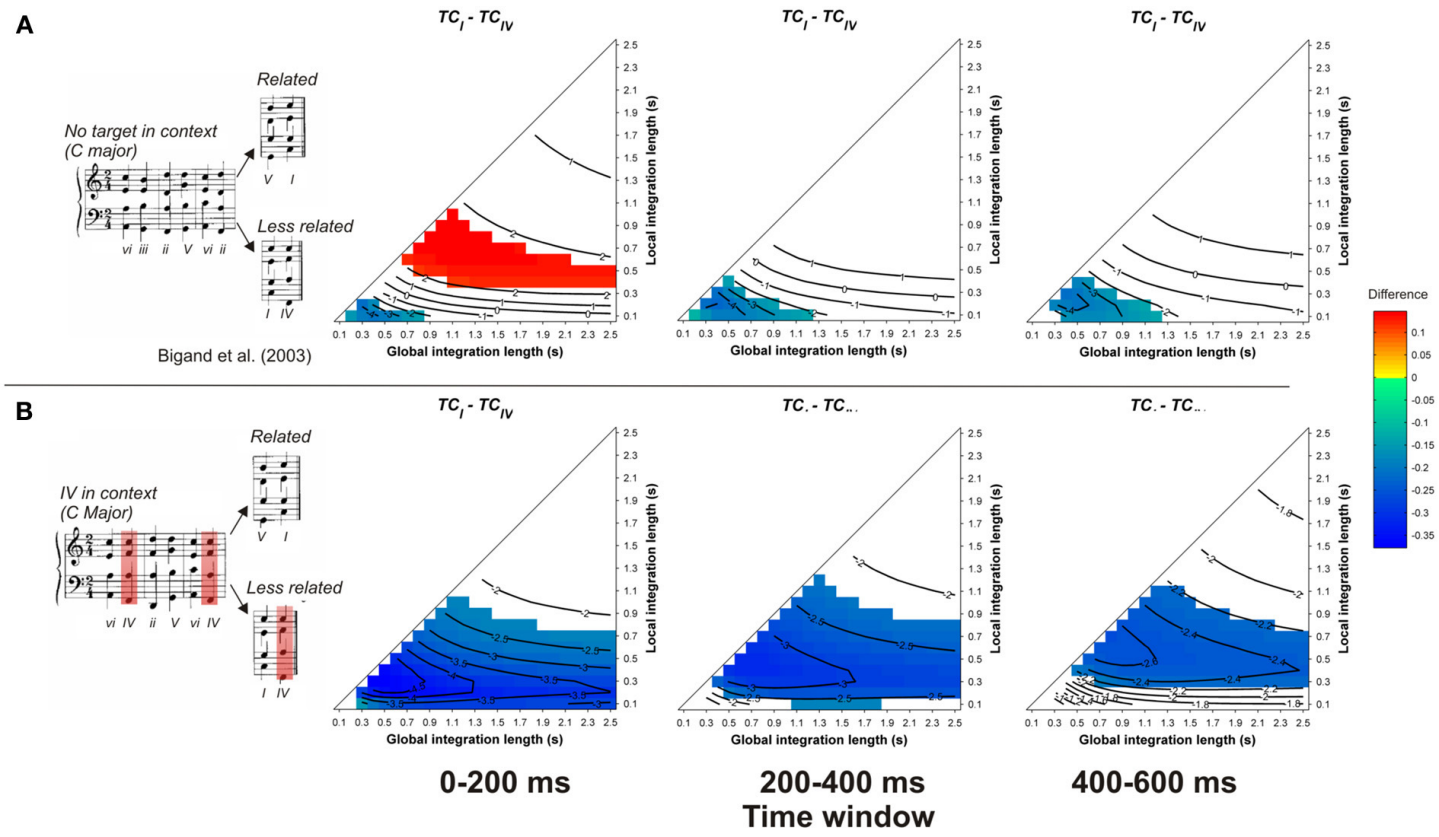
**Each row represents one experimental condition used by Bigand et al. (2003), in which the less-related target chord appeared in the context (B) or not (A)**. Example sequences of each condition are given in *inserts*. Each column represents the mean differences between *TC*_*related*_ and *TC*_*less_related*_ for a different time window post-target onset, as a function of the local and global context integration windows. Positive, negative, and nonsignificant differences are represented by hot, cold, and white colors, respectively (two-tailed paired *t*-test, *p* < 0.05; *t*-values are reported as contours).

In contrast to the preceding studies, the model fails to account for the observed priming effects in Bigand et al. (2003, see Figure 5). Whatever the memory parameters used and whatever the considered time window, the *TC* of the less-related target was higher than the *TC* of the related targets, whether the less-related target appeared in the context (Figure 5B) or not (Figure 5A). This difference was particularly strong for the condition where the less-related target appeared in the context, hence sharing numerous pitches with this context. This is the second example (over a total of 13 re-evaluated harmonic priming experiments) where the ASTM model cannot account for the data, thus suggesting that a cognitive account of these data may be plausible. In summary, this set of simulations suggests that the context dependency of chord function can be accounted for, almost entirely, by the ASTM model.

## Simulations of the ERP responses to violations of harmonic regularities

In ERP research, violations of musical syntactic-like structures have been reported to evoke late positive components as well as early negative components, which both have been compared to components evoked by violations of linguistic structures.

### Simulations of late positive components (LPC)

**Figure 6:**
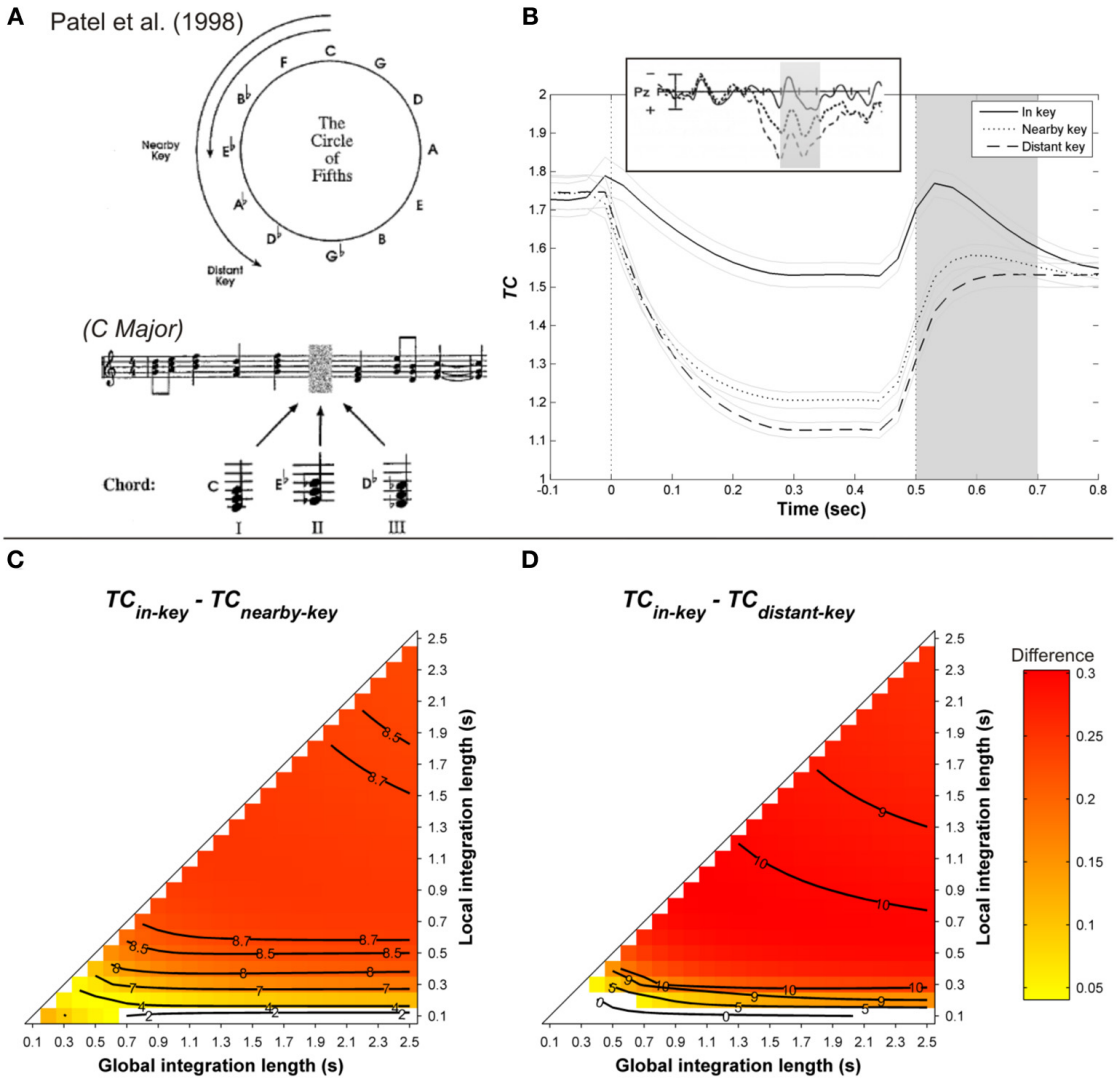
**(A)** Example of harmonic sequences used by Patel et al. (1998). **(B)** Mean running *TC*(*t*) of the target chord, as a function of its distance with the harmonic context (in-key, nearby, and distant-key: solid, dashed, and dotted line, respectively), computed with a local and global context integration windows of 0.3 and 1.5 s, respectively. Light gray lines represent standard errors. Insert: corresponding ERP responses. Gray area is the time window of the P600. Mean *TC* differences during the considered time window between in- and nearby-key targets **(C)**, in- and distant-key **(D)**, as a function of the context integration length. Positive, negative, and nonsignificant differences are represented by hot, cold, and white colors, respectively (two-tailed paired *t*-test, *p* < 0.05; *t*-values are reported as contours).

Janata (1995) and Regnault et al. (2001) observed that a LPC peaking around 300 ms post-onset was larger when a harmonically unrelated event occurred in the musical sequence. To investigate the late positive component, Patel et al. (1998) manipulated the strength of musical syntactic-like violations at three levels and compared the observed brain responses with those obtained with violations of syntactic structures in spoken sentences. The musical syntactic relation was manipulated in such a way that the target chord was either the tonic of the main key (in-key condition, syntactically correct), the tonic of a nearby key (nearby-key condition, syntactically incorrect), or the tonic of a distant key (distant-key condition, syntactically very incorrect, see Figure 6A). The local context surrounding the target, as well as the frequency of occurrence of the target in the previous context, were controlled. Similarly, sentences were either syntactically correct, or exhibited a moderate or a strong syntactic violation. The critical point of Patel et al.'s (1998) finding was to show a P600 for both linguistic and musical violations, the amplitude of which increased with the strength of the syntactic violation (see Figure 6B, *insert*).

As illustrated in Figure 6B, the ASTM model accounts for the P300 reported by Regnault et al. (2001). For the simulation of Patel et al.'s (1998) experiment, original MIDI sequences were used to synthesize the stimuli with a piano timber (an example of such sequences is given Figure 6A)^3^. The tempo was fixed at 120 beats/min, and the chord duration ranged from a 16th note (1/16 of a beat) to a tied halfnote (over 2 beats), in order to create rhythmic variety. As shown in Figures 6C,D, the effect of harmonic relatedness on the amplitude of the ERP component (Patel et al., 1998, see *insert* in Figure 6B) is also accounted for by the ASTM model: the differences for both “in-key *minus* nearby-key” and “in-key *minus* distant-key” comparisons were globally positive over the parameter space, indicating that the in-key target elicited the weakest acoustic dissimilarity, compared to both nearby- and distant-key targets. Moreover, these differences were stronger for the “in-key *minus* distant-key” comparison than for the “in-key *minus* nearby-key” one, which means that the stronger the music syntactic violation, the stronger the acoustic dissimilarity in the ASTM.

In addition, Figure 6B illustrates that the model responds in a continuous manner, as does the listener ^4^. In sum, the ASTM model predicts the effect of harmonic relatedness on the late component of the ERPs as observed by Patel et al. (1998).

### Simulations of the early right anterior negativity (ERAN)

An effect of harmonic relatedness on an earlier ERP component was also reported by Patel et al. (1998). Harmonic violation was associated with a Right Anterior Temporal Negativity, peaking around 300 ms after the onset of the unexpected target chord. In a series of studies, Koelsch and collaborators also reported an early ERP component, named ERAN peaking around 150 ms after an incongruous musical event. The ERAN has been interpreted as a marker of syntactic violations in music that is reminiscent of the ELAN observed in language (Koelsch and Friederici, 2003). The ERAN was elicited by three types of harmonic violations, with each one being assessed here with the ASTM model (see below).

#### Simulations of the ERAN evoked by the neapolitan sixth chord

In a set of studies (Koelsch et al., 2000, 2002, 2003, 2004), a Neapolitan sixth chord was used as the unrelated target. This chord is analyzed in music theory as a substitution of the subdominant chord, which is usually followed by a dominant seventh chord. The critical feature of interest of this chord is thus to instill a contextual acoustic dissonance (because of the two out-of-key notes), while being syntactically correct when used instead of a subdominant chord. A cognitive approach would predict a stronger disruption caused by this chord when used in a syntactically incorrect context compared to a syntactically correct context. By contrast, an ASTM-based approach would always predict a surprising effect of this chord, due to acoustic dissimilarity. If both acoustic and syntactic influences combine, we may expect a weaker effect of the Neapolitan sixth chord when occurring in a syntactically correct position compared to an incorrect position.

In Koelsch et al. (2000), five-chord sequences were used. The Neapolitan sixth chord occurred either at position 3, instead of a subdominant chord (syntactically correct position) or at position 5, where it replaced the tonic chord (syntactically incorrect position). As a control condition, the authors used a half-tone cluster (i.e., a dissonant group of four simultaneous tones that did not define a chord in Western tonal music), at positions 3 and 5. As the tone clusters corresponded to a strong unsyntactic dissonance, no effect of position was expected. Finally, the effect caused by syntactically incorrect chords was compared to the effect provoked by a change in timber (i.e., harmonic spectra of the chord). The main outcome was that all unexpected events evoked an ERAN, peaking at around 150 ms after the onset of the target event, even for the dissonant, but syntactically correct Neapolitan sixth (in position 3). A deviance in timber (change in instrument) also elicited a (strong) early negativity (around 150–200 ms post-onset). The critical point of the study was to analyze the amplitude modulations of this ERAN. The amplitude of the ERAN elicited by both Neapolitans and dissonant half-tone clusters was stronger on position 5 than on position 3. This effect of position on the ERAN was stronger for the Neapolitan targets. The observation that the ERAN was less pronounced at a syntactically correct chord position compared to an incorrect one could provide some evidence for some syntactic-like processing in music. However, this effect of position was also observed for unsyntactic tone clusters, and the ERAN was also found for timber deviance.

More recently, Leino et al. (2007) further investigated an effect of position using similar musical stimuli. The authors used seven-chord sequences, in which the Neapolitan sixth occurred at positions 3 or 7, where it replaced a tonic chord (syntactically incorrect configuration) or at position 5 where it replaced a subdominant chord (syntactically correct configuration). The effect of the Neapolitan sixth was expected to be stronger at positions 3 and 7 compared to position 5. In addition, Leino et al. (2007) used slightly mistuned chords instead of Neapolitan chords to compare syntactic violations with scale/tuning violations; the later deviance should elicit a MisMatch Negativity (MMN; 150–250 ms latency), reflecting short-term encoding of physical regularities in the auditory domain (Winkler et al., 1996). The crucial prediction was that the amplitude of the MMN elicited by mistuned chords should not vary across chord positions, while the amplitude of the ERAN evoked by Neapolitan chords should be smaller at the syntactically correct position 5 than at the incorrect positions 3 and 7. ERAN and MMN amplitudes were not found to differ significantly and the ERAN was elicited even earlier than the MMN (235 vs. 270 ms). The important point was a significant interaction between the type of violation and position. The Neapolitan sixth chord elicited a smaller ERAN at position 5, compared to positions 3 and 7, while there was no effect of position for mistuned chords on the ERAN (see Figure 8, *insert*). These findings strongly suggested that the ERAN might be considered as a neurophysiological marker of syntactic-like processing in music. As stated by Leino et al. (2007): “[…] our results provide support for the presence of specific neural mechanisms processing the rules of harmony that can be separated from the processing of other musical rules by a brain response with a distinct latency and morphology” (p. 175).

Simulations were run with the musical stimuli of Leino et al. (2007). Stimuli were synthesized according to their description and the score provided in the original paper, using the same timber (piano). As in the experimental paper, each stimulus cadence consisted of six 600 ms long chords followed by one 1200 ms long chord. Twelve transpositions of each of the four sequences (in-key, Neapolitan chord at position 3, 5, and 7) were used. As in the experimental study, stimulus sequences in different keys and with different experimental manipulations were presented in random order to the model, in a continuous manner. Ten different random orders were used; echoic memory state was reset between each of the 10 runs, but not between each sequence.

**Figure 7:**
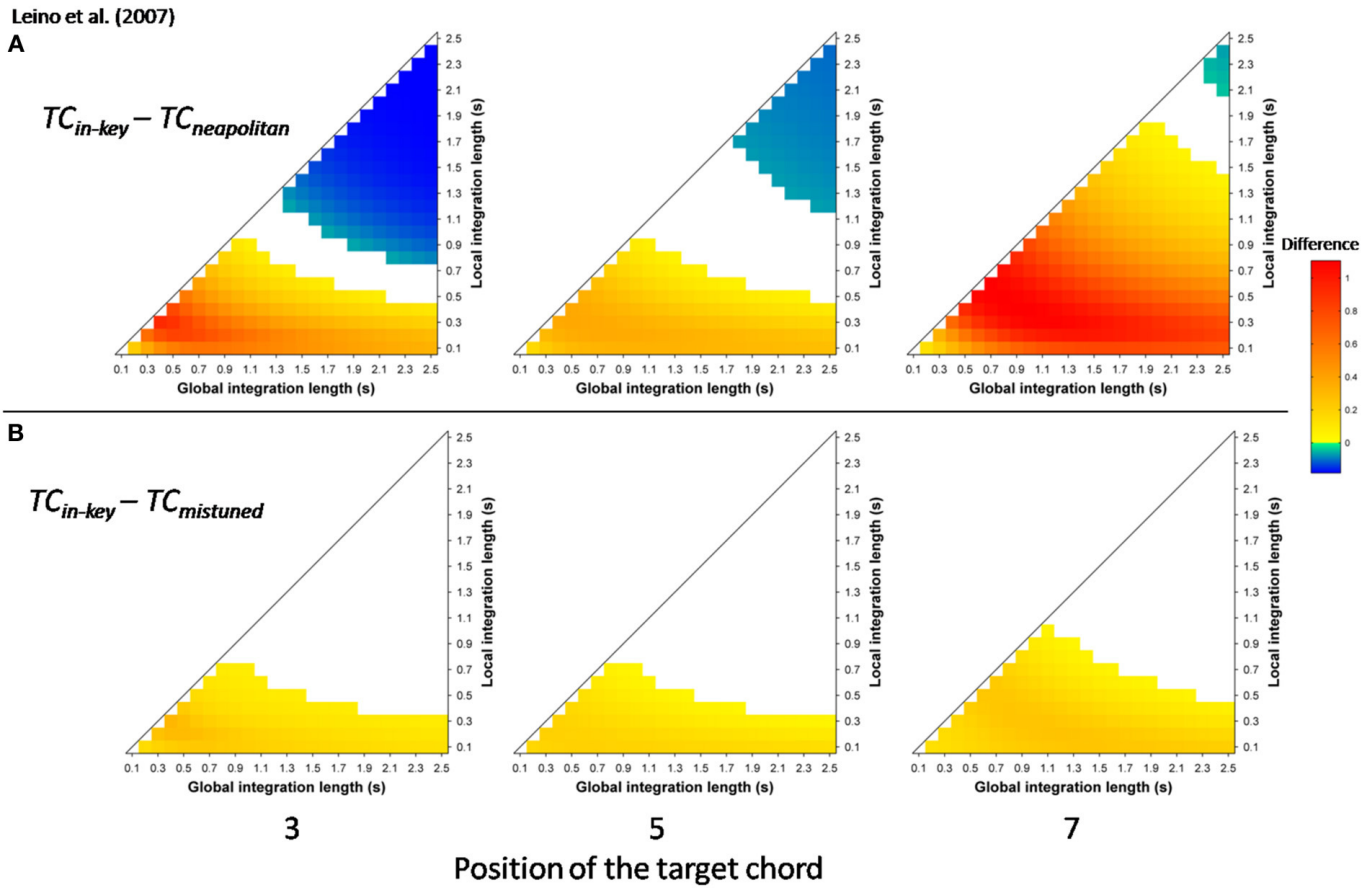
**Simulation data obtained with the stimuli of Leino et al. (2007)**. Mean *TC* differences (200 ± 25 ms after onset) as a function of the local and global context integration windows between in-key targets vs. Neapolitan chords **(A)**, in-key targets vs. mistuned (but in-key) chords **(B)**. Positive, negative, and nonsignificant differences are represented by hot, cold, and white colors, respectively (two-tailed paired *t*-test, *p* < 0.05; *t*-values are reported as contours). Each column represents a different position within the sequence.

Mean *TC* differences were computed for the window 200 ± 25 ms post-target onset, reflecting the latency of the ERAN observed in the electrophysiological study. The differences “in-key *minus* Neapolitan” chords (Figure 7A), and “in-key *minus* mistuned” chords (Figure 7B) were positive over the whole parameter space and at all positions. That is to say, all types of odd events elicited a stronger dissimilarity in ASTM than did the in-key target chords. In addition, the size of the dissimilarity mimicked the size of the ERAN. The dissimilarity was the strongest for the Neapolitan sixth (red color), being larger than the dissimilarity for the mistuned chords (yellow color). This pattern fits with the amplitude pattern of the ERAN reported for all these chords in the experimental study (Leino et al., 2007).

**Figure 8:**
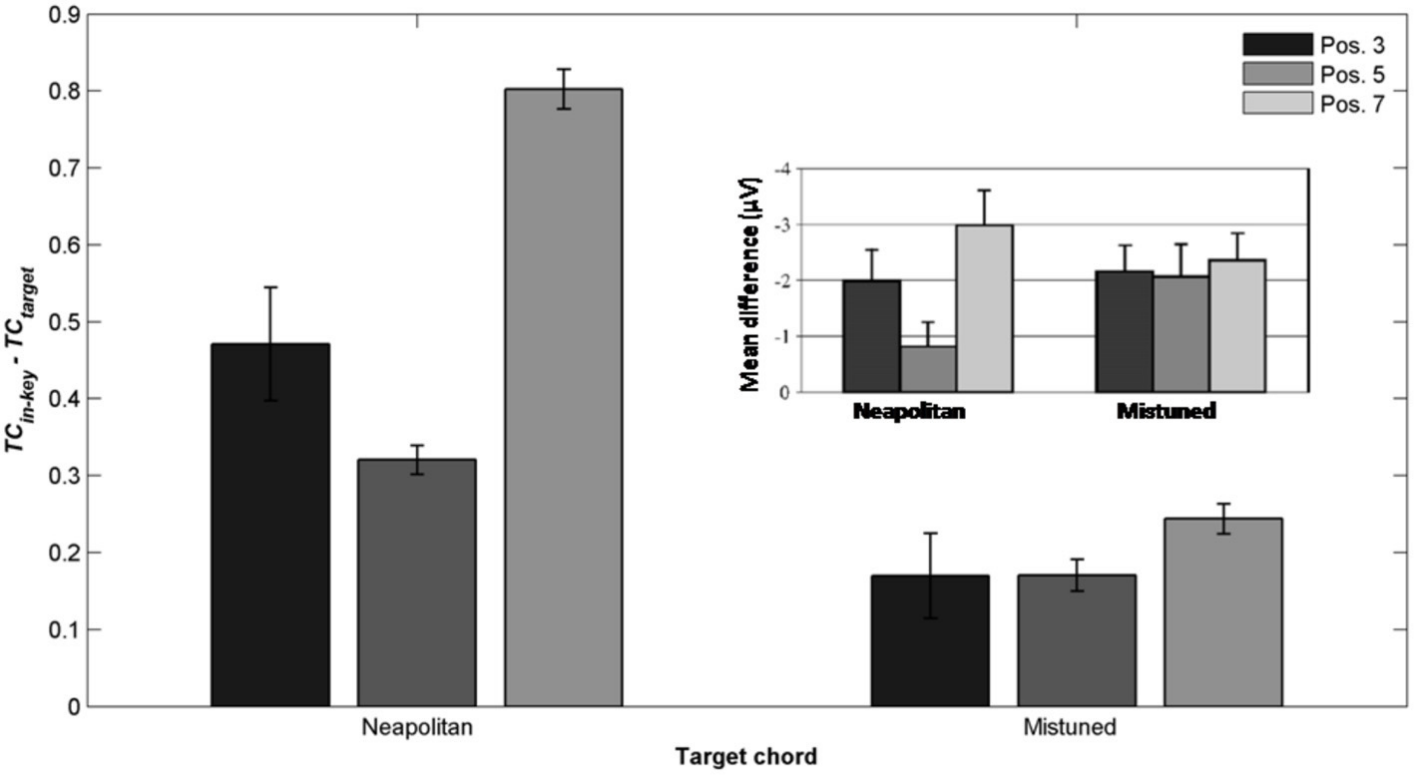
**Detailed simulation data obtained with the stimuli of Leino et al. (2007), computed with a local and global context integration windows of 0.1 and 1.5 s respectively**. Mean *TC* differences between congruent and incongruent target chords, as a function of the position of the target within the sequence, and of the condition. Insert: mean ERAN peak amplitude as a function of the position, for the Neapolitan and mistuned conditions, from Leino et al. (2007).

The most important point was to evaluate whether the ASTM model can account for the effect of position. Figure 8 shows the *TC* difference between in-key and Neapolitan chords in function of the chord position. Due to size limits considerations, we only reported this graph for the 0.1/1.5 s memory integration lengths (the set of memory parameters initially used by Leman, 2000). As illustrated Figure 8, simulations predicted an interaction with position, notably with stronger dissimilarity for the Neapolitan sixth occurring at the syntactically incorrect position, compared to the syntactically correct positions. In sum, the ASTM model accounts for the observed data pattern, suggesting that the effect of position on the ERAN might have an acoustic rather than a cognitive origin.

#### Simulations of the ERAN observed in bach chorales

The ERAN has also been reported with more refined harmonic manipulations. Steinbeis et al. (2006) manipulated syntactic-like harmonic violations within short excerpts of six Bach chorales. Each excerpt ended on either the expected tonic, a slightly unexpected minor chord substitution of the tonic, or a very unexpected Neapolitan sixth chord. An ERAN was observed for both types of unexpected events, peaking at about 200 ms for musicians and 230 ms for nonmusicians, with amplitudes that increased with the strength of the expectancy violations. According to the authors, the larger ERAN elicited by the very unexpected chords (compared to the slightly unexpected ones) reflects that these negativities are sensitive to the degree of harmonic expectancy violation. The interesting point of Steinbeis et al.'s (2006) study was to replicate with more realistic stimuli the findings initially observed with experimental stimuli (i.e., isochronous chord sequences). A further replication was recently provided by Koelsch et al. (2008), with similar syntactic-like manipulations performed in real musical pieces. A consistent ERAN was also reported for the unexpected events (i.e., a Neapolitan sixth chord).

**Figure 9:**
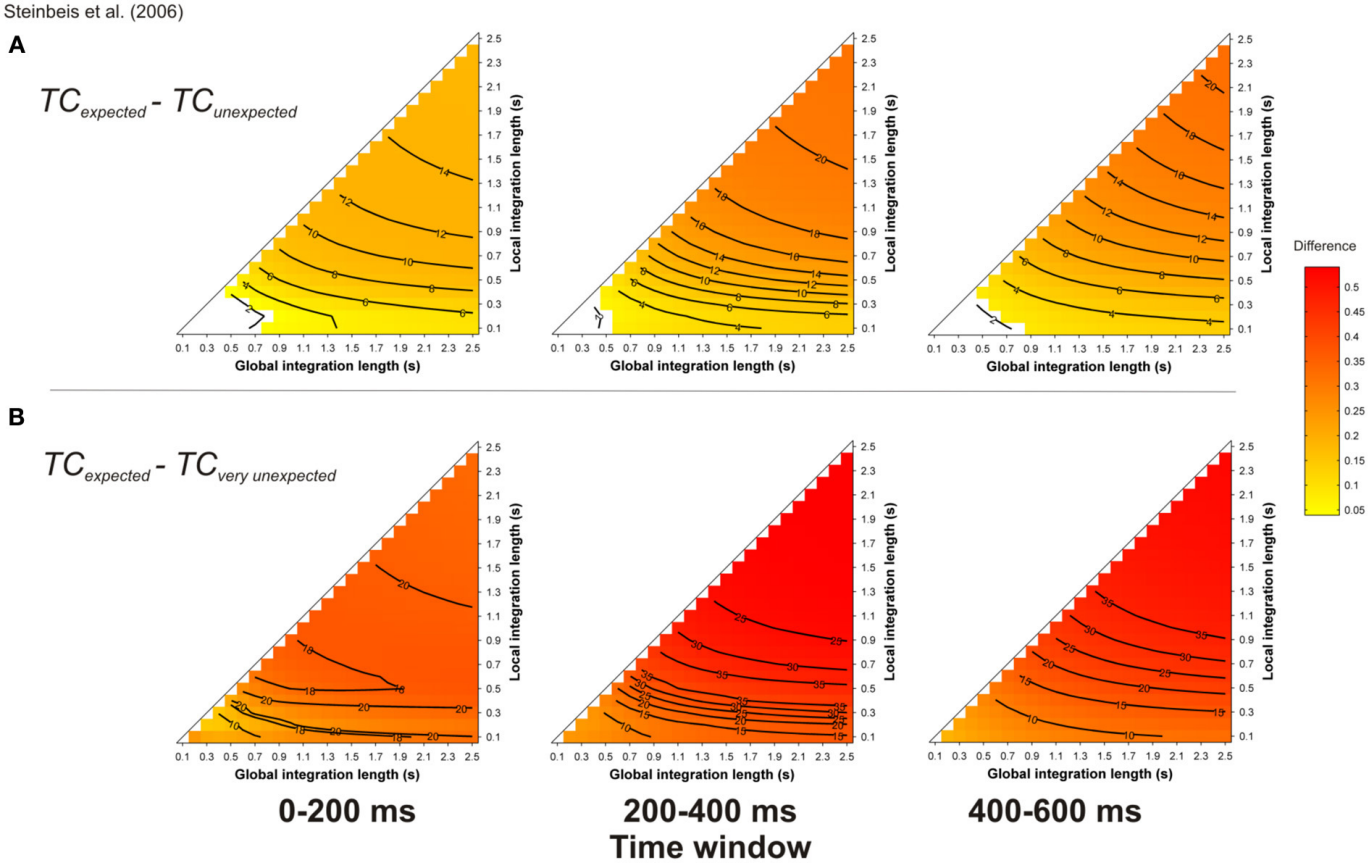
**Simulation of Steinbeis et al. (2006)**. Mean *TC* differences between expected vs. unexpected **(A)** and vs. very unexpected **(B)** target chords, as a function of the local and global context integration windows. Positive, negative, and nonsignificant differences are represented by hot, cold, and white colors, respectively (two-tailed paired *t*-test, *p* < 0.05; *t*-values are reported as contours). Each column represents a different time window post-target onset (note that the ERAN occurs in the 0–200 ms time-window).

Simulations were performed with the musical stimuli described in Steinbeis et al. (2006, their Figure 2), transposed in the 12 major keys, with a piano timbre. Figure 9 reports the mean *TC* differences between expected *minus* unexpected (A) and expected *minus* very unexpected (B) targets, computed during three different time-windows, 0–200, 200–400, and 400–600 ms post-onset. Note that the ERAN occurs in the 0-200 ms time-window. The pitch dissimilarity was stronger for unexpected (A) and very unexpected (B) chords compared to expected targets, as illustrated by warm colors. Furthermore, the *TC* difference between very unexpected and expected chords was stronger than the *TC* difference between unexpected (i.e., relative minor chords) and expected chords.

**Figure 10:**
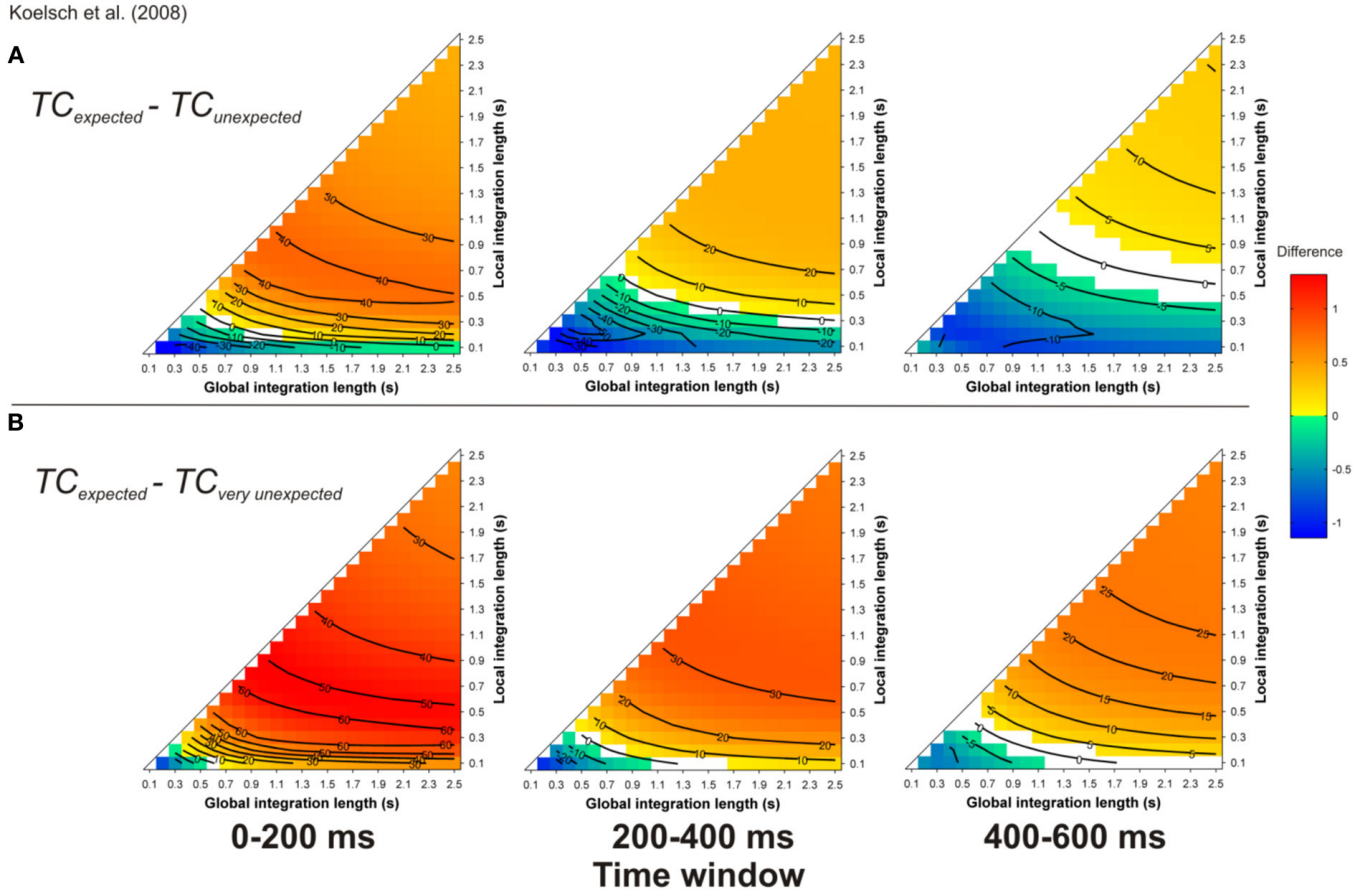
**Simulation of Koelsch et al. (2008)**. Mean *TC* differences between expected vs. unexpected **(A)** and vs. very unexpected **(B)** target chords, as a function of the local and global context integration windows. Positive, negative, and nonsignificant differences are represented by hot, cold, and white colors, respectively (two-tailed paired *t*-test, *p* < 0.05; *t*-values are reported as contours). Each column represents a different time window post-target onset (note that the ERAN occurs in the 0–200 ms time-window).

Simulations carried out for the more realistic material given in Koelsch et al. (2008) led to a similar finding. For most of the parameter space (but not all parts, compare cold and hot colored areas), the TC was stronger for expected targets (Figure 10A) compared to unexpected and very unexpected (Figure 10B) chords. Moreover, the TC was stronger for unexpected compared to very unexpected chords (particularly for 0–200 ms time-window), thus predicting the difference in amplitude of the ERAN elicited by these two chords.

#### Simulations of the ERAN evoked by the secondary dominant

**Figure 11:**
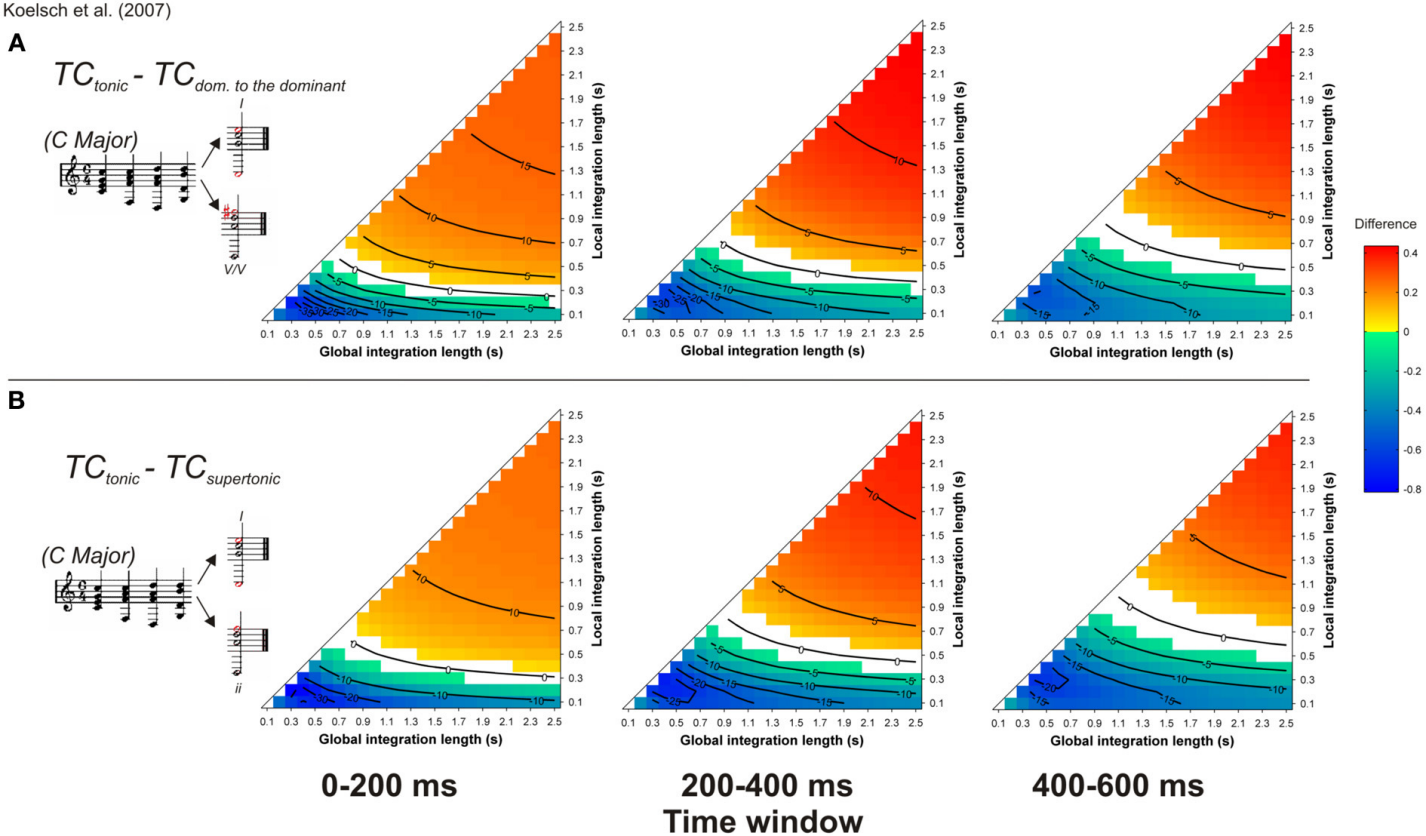
**Simulation of Koelsch et al. (2007)**. Mean *TC* differences between tonic and secondary dominant **(A)** and supertonic **(B)** target chords, as a function of the local and global context integration windows. Positive, negative, and nonsignificant differences are represented by hot, cold, and white colors, respectively (two-tailed paired *t*-test, *p* < 0.05; *t*-values are reported as contours). Insert: Koelsch et al. (2007) chord sequences. Each column represents a different time window post-target onset (note that the ERAN occurs in the 0–200 ms time-window).

Another type of syntactic violation was used to disentangle ASTM effects from cognitive effects of the ERAN (Koelsch et al., 2007; Koelsch and Jentschke, 2008; Koelsch and Sammler, 2008). In Koelsch et al. (2007), five-chord sequences were used in which the last chord was either a tonic, a supertonic (i.e., a minor chord which belongs to the key, but is syntactically less important than the tonic) or a secondary dominant (i.e., the dominant-to-the-dominant; a chord that belongs to another key, Figure 11, *inserts*). In terms of Lerdahl's (2001) TPST, the secondary dominant chord has a greater distance from the tonic than the supertonic minor chord. A syntactic model would predict a larger ERAN for the secondary dominant compared to the supertonic minor chord. The interesting new aspect of this material was that the most strongly syntactically related chord (the tonic) shared less component tones with the preceding context than did either the supertonic minor chord or the less-expected secondary dominant chord. The authors used the ASTM model of Leman (2000) to ensure that the tonic chord was creating the strongest acoustic dissimilarity, and accordingly, the ASTM model would predict a stronger deviance for the tonic than for the other chords. In contrast to the predictions of the ASTM model, both types of unsyntactic targets elicited an ERAN compared to the tonic chord. This ERAN was maximal around 200 ms after the onset of the chord and its amplitude did not differ between supertonic and secondary dominant chords. It was argued that because the supertonic did not deviate in terms of the number of the shared tones as well as pitch periodicity overlap, the ERAN could be interpreted as a marker of abstract music-syntactic processing.

The simulations reported by Koelsch et al. (2007) were performed with set values of 0.1 and 1.5 s, for local and global pitch integration windows, respectively. Our present simulations were performed with all possible values of the parameter space within the range [0.1 2.5] s, as presented above. This approach allowed us to assess whether the predictions made by the ASTM model were always in favor of the syntactically less-expected chord, or if these predictions changed for some parameter combinations. Stimuli were synthesized according to the description provided by Koelsch et al. (2007, their Figure 2, p. 478). As in the experimental study, piano timber was used, chords duration was 600 ms, and the target (last) chord sounded for 1200 ms. Figure 11 reports the mean *TC* differences between tonic *minus* secondary dominant (A) and supertonic (B) targets, computed during three different time windows, 0–200, 200–400, and 400–600 ms post-onset.

For both unexpected targets, the sign of the differences with the tonic chord depended on the used parameters: half of the parameter space predicted stronger *TC* for expected targets and the other half predicted stronger *TC* for unexpected targets. When the difference was positive (i.e., red colors), hence indicating a higher *TC* for expected targets, the effect was more pronounced at the beginning of the chord (first 200 ms window), which would fit with the latency of the ERAN. However, the interpretation of the simulations with this material depended entirely on the chosen memory parameters. Very short local integration windows led to the blue areas of Figure 11, suggesting that the ERAN reflects a purely syntactic-like computation. In contrast, for longer integration windows, ASTM suffices to account for the data, as indicated by the red colored areas. This is the third example of musical stimuli for which the ASTM model encountered some difficulties. Although some experimental data on the duration of ASTM suggest a duration of about 1 s, with a maximum of about 2 s for periodic noise (Guttman and Julesz, 1963), current knowledge about the dynamic aspects of ASTM is not precise enough to choose the psychologically relevant values of the memory parameters in the model. For now, the simulations thus simply indicate that the used stimuli do not allow concluding with certainty that the performed musical violations solely tap into syntactic-like processing.

#### Simulations of the ERAN evoked by violations of newly acquired artificial musical grammars

As we (and others, e.g., Parncutt and Bregman, 2000) have pointed out, the investigation of the respective influences of syntactic processing and of ASTM in Western tonal music processing is difficult because acoustic features cannot be fully isolated from learned syntactic regularities. One possibility to investigate this issue is offered by new or artificial musical languages that are based on different scale systems. An ERP study by Loui et al. (2009) exposed participants to short harmonic sequences generated from a previously unheard musical scale. While the Western scale divides an octave interval (a 2:1 frequency ratio) in 12 pitches, the Bohlen-Pierce scale (Mathews et al., 1988) divides a tritave (i.e., a 3:1 frequency ratio) in 13 constituents; this structure produces very unusual intervals between tones. The experiment consisted in presenting a standard sequence of four chords, each made of three frequencies approximating a simple ratio of 3:5:7, one of them being always shared with the previous chord. The standard sequence started on one of these three frequencies, and was presented 700 times. For 100 additional trials, the amplitude envelope of one chord of the standard sequence was changed (i.e., introduction of a fade-out). Finally, 200 other deviant sequences were included: here, one chord of the standard sequence was substituted by a new chord, which also shared one frequency component with its predecessor. Over a 1 h period of listening to this new musical system, ERPs elicited in a less probable sequence showed the emergence of an ERAN. As these negativities were eliminated when sound patterns were played equiprobably, the authors concluded that the ERAN indicated that participants learned the statistical regularities of the four-chord sequences.

**Figure 12:**
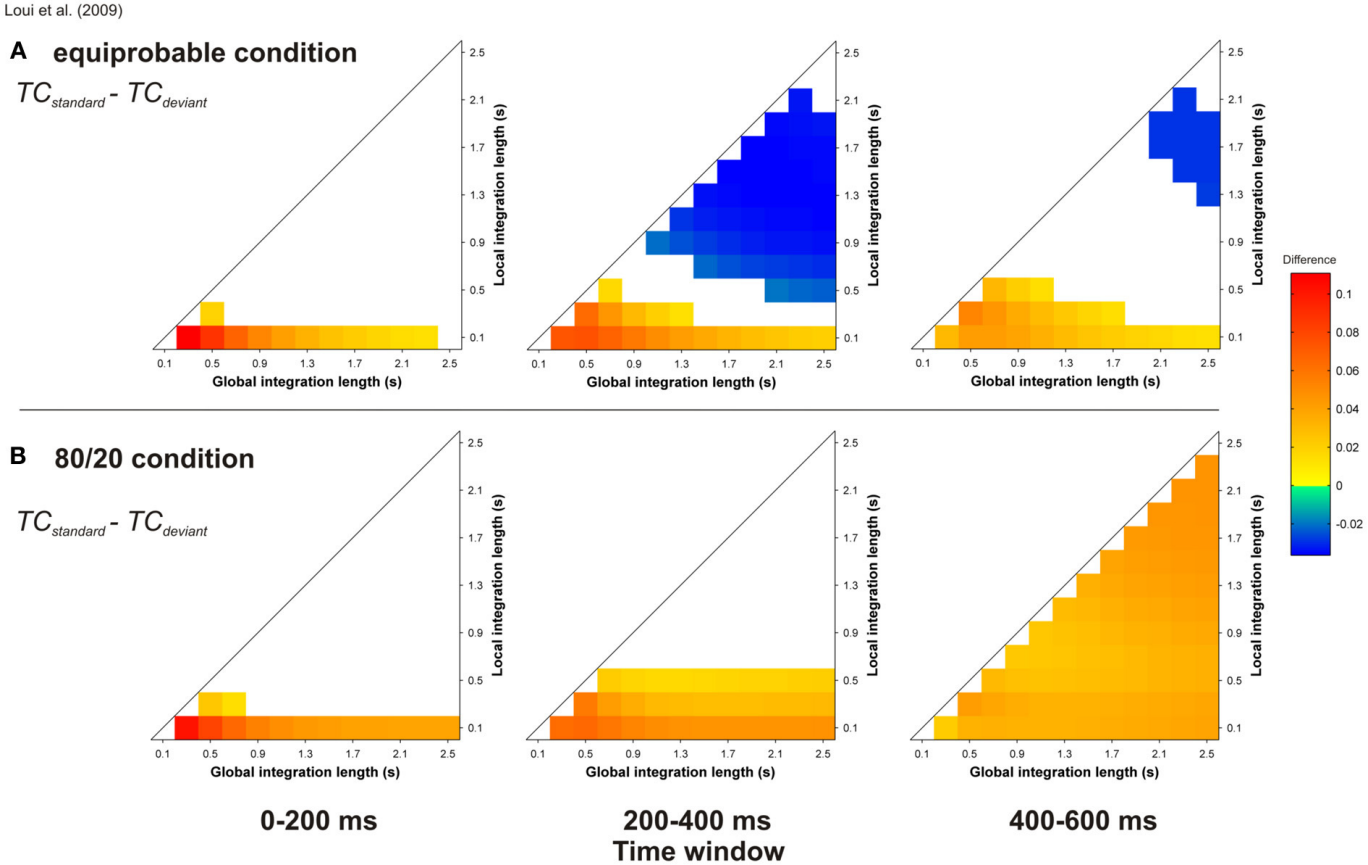
**Simulation of Loui et al.'s (2009) study**. Mean *TC* differences between standard and deviant target chords for the equiprobable condition **(A)** and for the 80/20 condition **(B)**, as a function of the local and global context integration windows. Positive, negative, and nonsignificant differences are represented by hot, cold, and white colors, respectively (two-tailed paired *t*-test, *p* < 0.05; *t*-values are reported as contours). Each column represents a different time window post-target onset (note that the ERAN occurs in the 0–200 ms time-window). In the present simulation, local and global integration lengths were computed with steps of 0.2 s, in comparison with the others (0.1 s).

Thanks to the unconstrained nature of the input to the ASTM model, we were able to conduct simulations for the material of Loui et al. (2009). As described in this article, chord duration was 600 ms, and 100 harmonic sequences were presented to the auditory model, with either an equiprobable occurrence of the standard and deviant sequences, or 80 % of standard and 20% of deviant sequences. These sequences were presented to the model in a random order, and the state of the ASTM was not reset between sequences. In order to ensure that the obtained result would not be due to a specific ordering of the sequences, 10 runs were independently computed and averaged. Figure 12 displays the *TC* differences for “standard *minus* deviant chords” comparison, for the equiprobable condition (panel A) and for the 80/20 condition (panel B).

The probability of occurrence of the deviant sequences compared to the standard sequences has a strong effect on the behavior of the model. When the standard sequence appeared more often than the deviant sequence, the mean *TC* of the target chord was higher for the standard compared to the deviant, while this difference for the equiprobable condition was only observed for smaller areas of the parameter space. Thus, the ERAN elicited by deviant stimuli over a period of an hour could be merely due to the accumulation of pitch images in ASTM, rather than to the effect of an emergent abstract knowledge of syntactic-chord progressions.

In sum, the present simulations allow questioning the cognitive nature of the ERPs elicited by the occurrence of musical unexpected events. The influence of the factors that were shown to modulate the amplitude of the ERPs (i.e., the harmonic relatedness of a target chord with the musical context and the position of this target chord within the harmonic sequence) were accounted for by the ASTM model. A parsimonious interpretation of the reviewed neuroscientific data thus suggests that the observed ERPs might be caused by an acoustic dissimilarity between the auditory input of the unrelated target and the auditory information of the context stored in ASTM.

## Simulations of interfering effects between music and language processing

A group of experiments has directly addressed the overlap between music and language processing by reporting interfering effects between musical and linguistic processes. Previous studies have shown that manipulating the harmonic function of chords modulated the processing of sung phonemes (Bigand et al., 2001) and the size of semantic priming in sung music (Poulin-Charronnat et al., 2005). Interfering effects may reflect the influence of general attentional processes on musical processing, and they were found to be nonspecific to language, in particular the beneficial effects of the expected tonic chord (Tillmann et al., 2003b, 2008; Escoffier and Tillmann, 2008).

Some studies reported interfering effects between music and language and also added an experimental condition designed to disentangle specific interfering effects from general disruption of attention due to unexpected events (e.g., loudness or timber oddball). Koelsch et al. (2005) reported that the LAN elicited by syntactically incorrect words was reduced when words were presented simultaneously with music-syntactically irregular chords (Neapolitan sixth of Koelsch et al., 2000; see Figures 7–10). By contrast, the N400 elicited by semantically unrelated words was not affected by the occurrence of the Neapolitan sixth chord. When a single repeated tone (presented in an isochronous sequence) was used instead of chord sequences as a control condition, the introduction of a deviant tone influenced neither the LAN nor the N400 (compared to a standard tone). This finding provides some evidence that musical syntactic-like violation interferes with linguistic syntactic processing, but not with semantic processing. The specificity of this interfering effect was not replicated by Steinbeis and Koelsch (2008) in a very similar experiment: the same music syntactic-like manipulation affected both the size of the ELAN when syntactic errors occurred in sentences, and the size of the N400 when semantic incongruities occurred in sentences (note that no control condition was used). This finding was interpreted by the authors as demonstrating an overlap in music processing and semantic processing of language. To our view, a more parsimonious interpretation is that the *TC* of the Neapolitan sixth chord is lower than the *TC* of the tonic chord (see Figure 7), and that this lower *TC* instilled by the Neapolitan sixth chord disturbed attentional processes that resulted in modulating the responses to both syntactic and semantic errors. However, the ASTM model used in our present study cannot investigate this attentional effect in a direct way.

Further behavioral experiments addressed the specificity of the interference caused by violations of musical syntax. In Slevc et al. (2009), musical syntactic violations very similar to those of Patel et al. (1998, see above) were used. Target chords and words were time-locked, and the occurrence of the syntactically incongruent chord corresponded to either the beginning of a garden path or the occurrence of a semantically unrelated word. The authors reported interfering effects of music on garden path effects but not on semantic violations, suggesting that the processing of the musical incongruity taps into the same integrative processes as syntax, but not as semantics (but see Hoch et al., 2011; Perruchet and Poulin-Charronnat, 2013). Fedorenko et al. (2009) used sung melodies in which an out-of-key tone instilled a syntactic-like violation. This out-of-key tone could co-occur with an increase in the complexity of the syntactic structure of the linguistic sentence. Interference effects were again reported between musical and linguistic manipulation. Moreover, no interfering effects were observed when the loudness of the tone was unexpectedly increased by 10 dB (Fedorenko et al., 2009), or when the timber of the target was changed (Slevc et al., 2009). These findings were taken as strong evidence for shared resources between musical and linguistic syntax processing.

**Figure 13:**
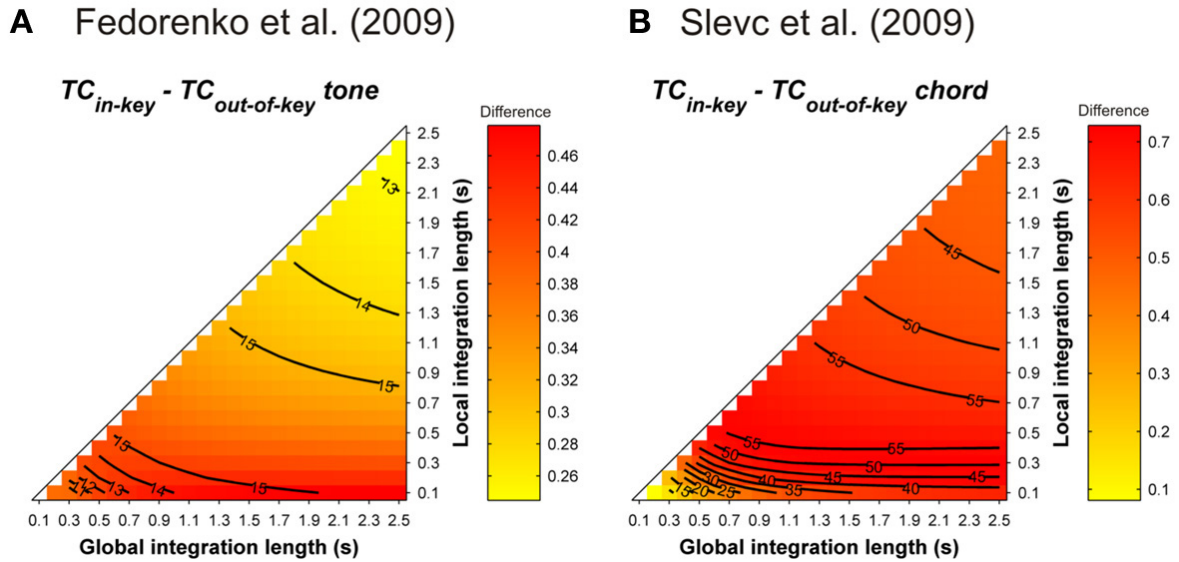
**Mean *TC* differences between tonic vs. out-of-key tones (A, Fedorenko et al., 2009) and tonic vs. out-of-key chords (B, Slevc et al., 2009), as a function of the local and global context integration windows**. Positive, negative, and nonsignificant differences are represented by hot, cold, and white colors, respectively (two-tailed paired *t*-test, *p* < 0.05; *t*-values are reported as contours).

Simulations were performed to assess the ASTM-based impact of the syntactic-like manipulations performed in the musical stimuli used in Slevc et al.'s (2009) and Fedorenko et al.'s (2009) experiments. Simulations used the sequence presented in Figure 1 of Slevc et al. (2009, p. 376, transposed in 12 major keys with a piano timbre, chord duration was fixed to 600 ms). For Fedorenko et al. (2009), tonal melodic sequences were generated from the authors' description. For each of the 12 major keys, 10 sequences of five tones randomly selected among the seven pitches of a given key were constructed. Tones sounded for 500 ms. Mean *TC* differences between tonic and out-of-key target tones following these sequences (Figure 13) showed that, whatever the temporal integration parameters, tonic chords/tones instilled a stronger *TC* with the melodic (Figure 13A) and harmonic (Figure 13B) contexts compared to out-of-key tones and chords.

The simulations showed that the violations involved in these musical manipulations were confounded with a strong contextual acoustic dissonance based on information stored in ASTM. Hence, interfering effects between music and language were not necessarily caused by the fact that listeners were processing the musical syntax at a cognitive level. An ASTM approach offers an alternative and more parsimonious explanation of the interfering effect: a strong dissimilarity in the tone sensations experienced by the listeners, due to an incongruous chord, could disrupt attention, which would results in interference with concurrent linguistic processing.

## General discussion

Even though there is a large consensus about the syntactic organization of Western tonal music, the very nature of the processes that are involved in music syntactical processing remains a matter of debate. According to some authors, musical and linguistic syntax processing tap into the same cognitive and neural resources (Patel, 2008), but numerous other authors have proposed that some aspects of syntax processing in music could be accounted for by an ASTM model (Deutsch, 1984; Parncutt, 1989; Huron and Parncutt, 1993; Leman, 2000). The present study further investigated this issue by revisiting a set of emblematic research data on musical syntax processing. Leman's (2000) model was proposed as a methodological tool for assessing the power of an ASTM model, even though this model does not integrate several other aspects of auditory processing such as stream segregation, roughness perception, and timbre. Given that all these studies were explicitly designed to address the cognitive processing of musical syntax rather than the (psycho-)acoustic level of auditory processes, this large set of data defines a considerable challenge for a rather simple ASTM model. Table 1 provides an overview of all simulations ran in the present study, and their main conclusions. The few cases for which the ASTM model encountered difficulties to account for the data are clearly identified (labeled as “cognitive account”).

**Figure 14:**
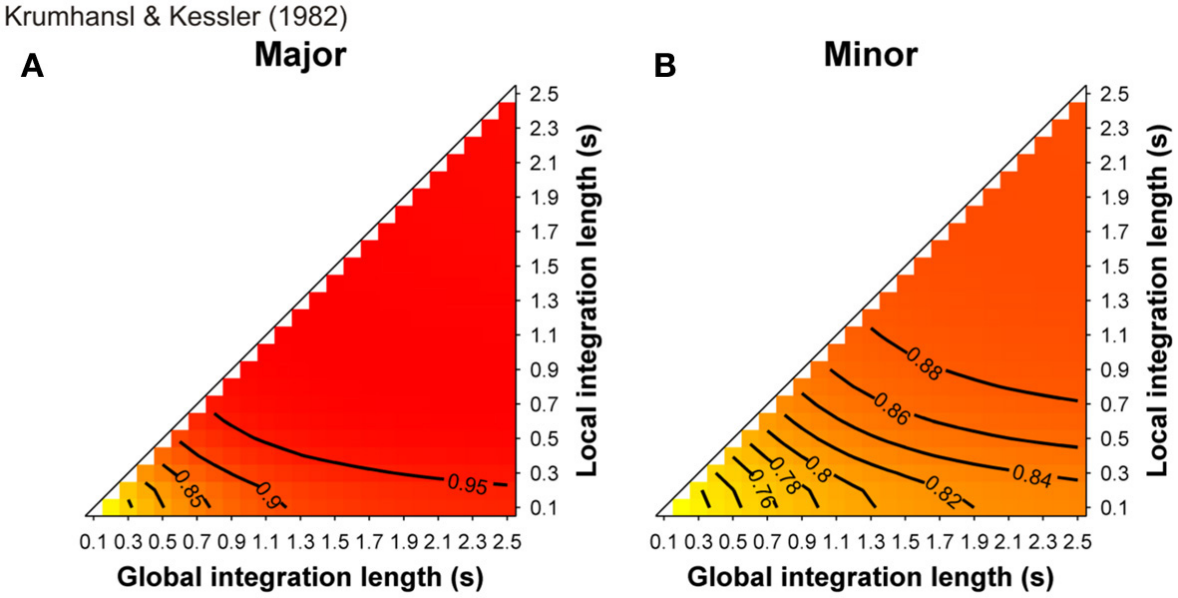
**(A,B)** Correlation values between the simulated tone profiles and the profiles obtained by Krumhansl and Kessler (1982) with the scale contexts of major and minor modes, respectively. *r* coefficients are reported as contours.

(1) The first contribution of our study is to provide new evidence that despite its limitations, a rather simple model of ASTM provides a good fit of a large set of studies that were explicitly designed to tap into cognitive levels of processing. The present simulations notably demonstrated that the context dependency of musical events could be an emergent property of ASTM. All scholars agree that the musical functions of tones and chords in Western tonal music depend on the musical context in which they appear. This context dependency is a core aspect of syntactic structures in music, and some authors considered that it provides “one strong piece of evidence for a cognitivist view of tonal syntax” (Patel, 2008, p. 260). The fact that long-term dependency between musical events as tested in the numerous studies simulated here can derive from ASTM is a new finding, which could not have been anticipated without the present simulations. An emblematic illustration is provided by the studies of Koelsch et al. (2000) and Leino et al. (2007). In both studies, the context dependency of a chord was manipulated by playing the same chord (Neapolitan sixth chord) at different positions within the sequence. At some position, this chord was syntactically possible, but not at others. Once again, the ASTM model simulated this context dependency. Our finding on chord sequences thus replicates and supports previous findings reported by Leman (2000) for tone perception and previously reported by, e.g., Huron and Parncutt (1993) as discussed above. According to Krumhansl (1990), the stability of tones in key contexts is the critical feature of the cognitive foundation of musical pitch. Leman (2000) provided a formal demonstration that the key profiles observed by Krumhansl and Kessler (1982) could be derived from ASTM (see also Huron and Parncutt, 1993). We replicated these simulations and extended them by using all possible pairs of the two free parameters and accounted for these data (Figure 14). All simulations reported in the present paper suggest that the rather simple and parsimonious model of ASTM for pitch can account for a large set of data, usually interpreted as demonstration of the cognitive nature of pitch processing. These findings do not rule out the possible contribution of cognitive processes to musical syntax but it provides a clear demonstration that this cognitive level of representation is not indispensible for listeners to respond to some syntactic-like organizations in music, and this even in experiments that were designed to specifically tap into this high level of processes.

It could be argued that the ASTM model failed to account for two empirical data sets (Tekman and Bharucha, 1998; Bigand et al., 2003; see Table 1), and provided an ambiguous account for two others (Koelsch et al., 2007, 2008; see Table 1). The apparent failure of the ASTM model for these studies can be interpreted in two ways. On the one hand, it points out some limitations of Leman's (2000) ASTM model, and indicates that this model needs to be improved. Indeed, the acoustical approach used in the present model combines a temporal model of pitch perception with a leaky integrator model of ASTM. The version of the auditory model that is used (Van Immerseel and Martens, 1992) does not take in consideration the role of place coding of pitch in the auditory system (for recent reviews of pitch theories and models, see, e.g., Yost, 2009; Oxenham, 2013, and of evidence of pitch representations in the brain, see, e.g., Bidelman and Krishnan, 2009). Furthermore, other psychoacoustic aspects of harmonic perception (such as voice leading and chord roughness) are not taken into account by the model. Whether the addition of these features to the model could possibly solve the ambiguity evoked by the musical stimuli used in these four studies (Tekman and Bharucha, 1998; Bigand et al., 2003; Koelsch et al., 2007, 2008) and could possibly simulate the collected human data patterns, remains a topic for further research. On the other hand, the failure of the ASTM model for just two studies, among the numerous other successfully simulated studies, can hardly be considered as a convincing demonstration that musical syntax processing involves linguistic syntactic-like computations. It simply suggests that other processes than those captured by Leman's (2000) ASTM model need to be added. Moreover, it is worth underlining that these two studies used indeed a peculiar material. Tekman and Bharucha (1998) used pairs of chords as stimuli. With such short auditory stimulations that manipulate rather subtle syntactic relations, a model of ASTM is the most likely to be in difficulty. The effect of accumulating information in memory would be more appropriately addressed with longer stimuli. Bigand et al. (2003) used longer chord sequences, but these sequences followed a specific construction in order to highlight cognitive contributions. Indeed, these sequences were composed in such a way that they contained numerous chord substitutions to reduce the occurrence of the related tonic target and to increase that of the less-related subdominant target in the prime context. These stimuli were syntactically legal, but weakly representative of the statistical distribution of chords that is usual in Western tonal music (Piston, 1978; Huron, 2006). As illustrated with the simulations of several other studies (e.g., Steinbeis et al., 2006, Figure 9; but see the ambiguous results with the study of Koelsch et al., 2008, Figure 10), the model performed better with musical stimuli bearing stronger similarities with everyday-life tonal music. This suggests that good performances of the ASTM model would be reached with more realistic music, rather than with short and artificial sequences.

(2) The present findings do not rule out the possible contribution of cognitive processes to musical syntax, but they provide evidence that the explanatory power of the ASTM may have been underestimated or misunderstood by the research community. The first implication of this finding relates to the methodology of future studies. Given the strong link between musical syntax and acoustical contextual information in ASTM, a direct comparison of musical and linguistic syntax processing should no longer be conducted without numerous controls of the experimental stimuli. Counting the musical tones that are shared by a given target and the preceding context, as done, for example, by Patel et al. (1998), Bigand and Pineau (1997) or even Tekman and Bharucha (1992), does not define a compelling control. The second implication of the present study is that the simulations run with the ASTM model should be done for all possible values of the decay parameters. Indeed, it may be the case that some musical sequences support cognitive priming for part of the model's parameters, while they support ASTM-based priming for the other part. As at present we do not know the exact decay values that are psychologically the most relevant, conclusions reported with such restricted simulations should be interpreted with caution. In a related vein, it can be stated that studying the interfering effects between the processing of linguistic sentences and musical sequences may open up promising avenues for the comparison of music and language. However, when the syntactic-like violations of the musical material create a strong acoustic disruption with the contextual auditory input, as the simulations demonstrated for Fedorenko et al. (2009) and Slevc et al. (2009), the very nature of this interference remains difficult to interpret.

Our study here outlines directions for the creation of new and more convincing manipulations of musical syntax. If music processing is sharing syntactic processes involved in language processing, then it should be possible to demonstrate that syntactic abilities in music are independent of changes in the acoustic information of the auditory input. One way to reach this goal with Western tonal music would be to assess the cognitive ability to differentiate a syntactically correct chord instilling strong pitch dissimilarity (called “dissonant” hereafter), from a syntactically incorrect chord. The interesting point would be to contrast the contextual pitch dissonance created by these chords with dissonance created by chords that violate the musical syntax. The single experiment that elegantly provided such a comparison between contextual pitch dissonance and syntactic dissonance created by chords that violate the musical syntax was the one of Koelsch et al. (2000). As described above, the Neapolitan sixth acted either as a syntactically correct chord at position 3, or as a syntactically incongruent chord at position 5. The most interesting point was to compare the effect of position for the Neapolitan sixth to the effect of position of a tone cluster, which creates acoustic dissonance without being syntactically correct. The brain responses to both manipulations were expected to reflect a differentiation between correct syntactic dissonance and incorrect acoustic dissonance. However, as stated by the authors, “the smaller amplitudes of ERAN and N5 elicited by Neapolitans at the third compared to the fifth position could not merely be due to the fact that Neapolitans at the third position are culturally more accepted (due to their function as a subdominant variation), since clusters also elicited smaller effects at the third position” (p. 537). This finding thus suggests that differentiating both types of dissonance (syntactic and acoustic) would not be such an easy task for the human brain.

(3) The explanatory power of the ASTM model contributes to shed new light on the cognitive foundation of human syntactic abilities in music and language. The present study suggests that syntax-related effects in music originate from ASTM processes, and Western listeners may thus respond to the Western musical syntax organization without having to rely on a mental representation of the rules of the Western musical syntax, the abstract musical function of events (e.g., tonic, dominant, subdominant), or even any abstract representation of tones, chords, and scales. We do certainly not deny that cognitive representations of tonal musical syntax may exist for music perception. Moreover, they are important in music performance, involving action and perception (e.g., jazz improvisation). However, the present simulations support the view that the cognitive level of representation as abstract as those existing in language is not a necessary condition to respond to musical syntax in music perception studies. In most of the situations we reanalyzed, the acoustic information accumulated in ASTM was in fact sufficient. Similar parsimonious explanations were already advanced for other syntactic-like processes. For example, research on implicit learning has provided strong evidence that participants may become sensitive to the regularities of a symbolic artificial grammar underlying sequences of stimuli without having any mental representation of that grammar, nor performing any syntactic-like computations (e.g., Perruchet and Pacteau, 1990; Perruchet and Vinter, 2002).

Rethinking musical syntax as a process that is rooted in ASTM may open new interesting theoretical perspectives. First, this approach could help to clarify certain findings in developmental research on tonality processing. For example, fMRI research on music processing in neonates suggested that “newborn's brain is sensitive to changes in the tonal key of music and to differences in the sensory dissonance of musical signals” (Perani et al., 2010, p. 4762). In this study, 3-day-old infants listened to three versions of a harmonized piano piece. The “control version” was a consonant piece obeying to Western musical rules. A first deviant version was derived by playing the two upper voices one semitone above their original pitches; a manipulation that created numerous highly dissonant pitch intervals. A second deviant version, more acceptable from a syntactic point of view, was performed by introducing several shifts in tonal key, one semitone above or below the main key. Using Leman's (2000) ASTM model (with the original memory parameters) for the second deviant version (but not for the first deviant version), the authors showed that these changes in tonal keys introduce a strong and sudden decrease in *TC*. Infants' brain responses to the control version differentiated from brain responses to the two deviant ones, which did not differ,. This finding suggests that the very first response to musical syntax originates from processes based on ASTM, as the computer simulation based on acoustic processing is able to distinguish between the control version (original tonal piece) and the second deviant (with shifts in key). The main conclusion of this study should thus be rephrased accordingly: “Newborn's brain is sensitive to changes in acoustic dissonance and to the changes in tonal contextuality consecutive to sudden changes in key.” In other words, Perani et al.'s (2010) findings showed that neural sensitivity to musical key can be parsimoniously explained based on acoustic information with the help of Leman's (2000) ASTM model.

**Figure 15:**
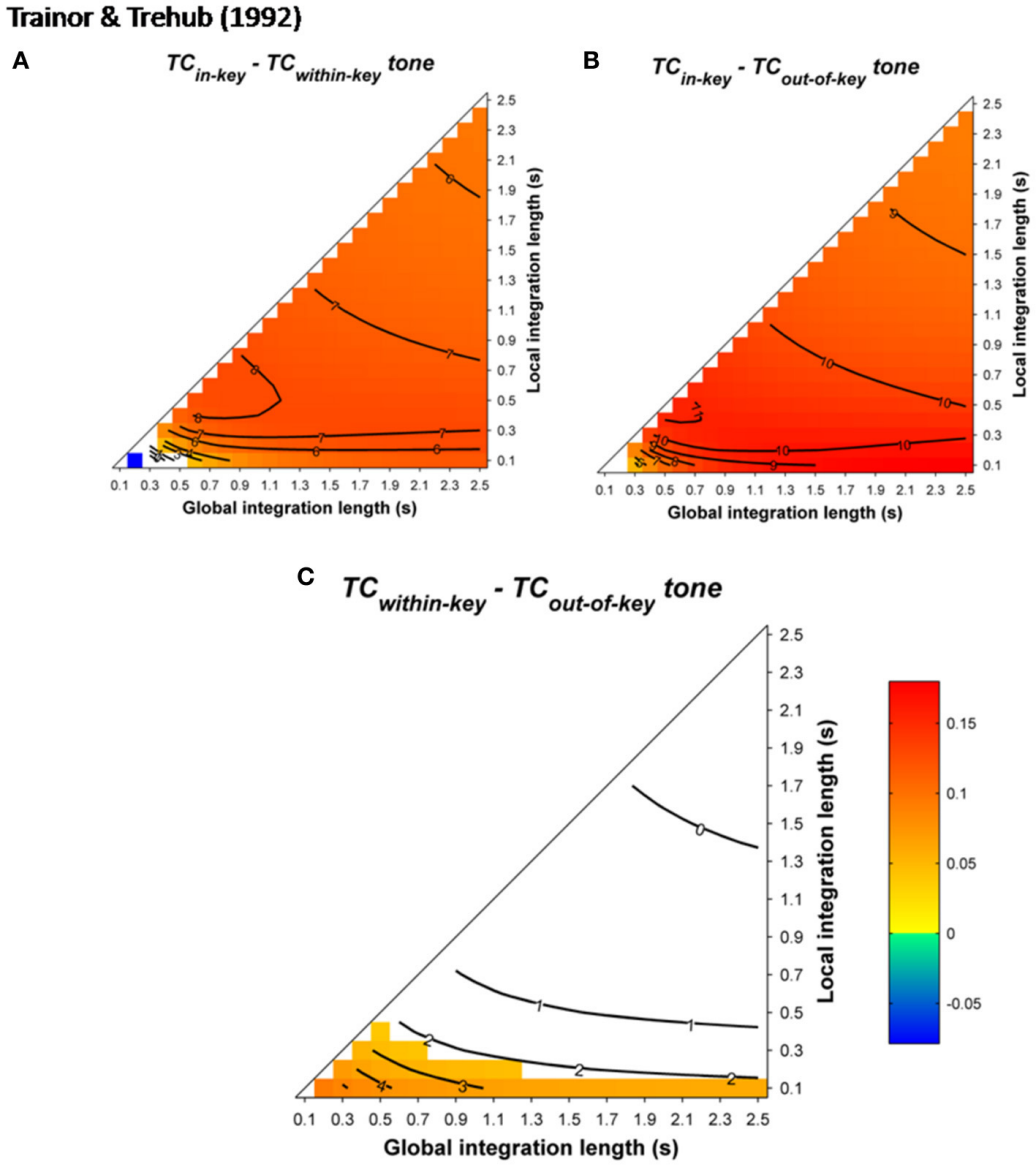
**Simulation of Trainor and Trehub's (1992) study**. Panels **(A,B)** show the *TC* differences for in-key *minus* within-key and in-key *minus* out-of-key targets, respectively. Positive, negative, and nonsignificant differences are represented by hot, cold, and white colors, respectively (two-tailed paired *t*-test, *p* < 0.05; *t*-values are reported as contours). All differences are positive (red) for both targets, but out-of-key targets are less correlated with the context than within-key targets (the map in **B** is redder than in **A**). This is illustrated in panel **(C)** that shows the difference of *TC* between within-key minus out-of-key targets. When significant, these differences are positives, meaning that *TC*_within-key_ > *TC*_out-of-key_. In other words, the out-of-key tone decorrelates more than the within-key tone. The white area indicates no significant difference between both targets.

The predominance of acoustic processing in infants' perception of musical syntax is not a surprising finding. However, the fact that this processing level allows neonates to respond to some aspects of musical syntax (distant key modulations) is rather a new finding that raises a further question: how does the human mind go from acoustic processing to syntactic processing in the domain of musical syntax during the course of development? In Trainor and Trehub (1992), 6-month-old infants showed equal sensitivity to in-key and out-of-key changes in a Western tonal melodies. By contrast, Western adults more easily detected out-of key changes in the same melodies. On the one hand, this difference in performance could be easily interpreted as a demonstration of a change occurring during development to reach a cognitive level of representation. On the other hand, the maturation of the infant brain is likely to considerably modify even low-level perceptual processing during development, including ASTM, as attested by the comparisons of MMN components in infants and adults (He et al., 2009; Trainor and Corrigall, 2010). Accounting for such differences in perceptual responses of infants and adults to Western pitch structures defines a further interesting challenge for the ASTM model. Would this model simulate infants' perception or adults' perception? To address this issue, Trainor and Trehub's (1992) pure tone melodies were generated, transposed in the 12 keys and then presented to the model. The *TC* of the in-key and out-of key tones was computed. Not surprisingly, the *TC* of in-key and out-of-key tones was lower than the *TC* of the original tone (Figures 15A,B). This suggests that for both infants and adults, a change in pitch in a short melody can be detected by its lower *TC*. The critical finding was that the model mimics infants' data for half of the decay parameters: both in-key and out-of-key changes led to an equal *TC* difference with the original tones (Figure 15C). These parameters correspond to a slow integration of the target tone into the ASTM. However, for another set of decay parameters, the model mimics the adults' data, with a greater difference in *TC* value between the out-of-key and original tones. These parameters correspond to a faster integration of incoming sounds at the local level in the ASTM. This simulation leads to the new suggestion that changes in dynamic properties of the ASTM could account for some developmental changes in pitch structure perception as reported by Trainor and Trehub (1992). Moreover, the differences in decay parameters for infants and adults indexed by the model are roughly compatible with functional changes that occur during infancy in ASTM (Trainor and Corrigall, 2010).

Given that the ASTM model rests on a very fundamental mechanism of temporal integration, the previous conclusion drawn from the discovery of new musical syntax processing in infants should be transferable to any situation where listeners are faced to a new musical idiom. To some extent, the simulation of Loui et al.(2009, see Figure 12) provides a first investigation of that issue. Using a new musical system obeying to a new tuning to define an artificial syntax that did not overlap at the acoustic level with Western music, this study provided evidence that Western listeners managed to internalize the regularities in sound induced by this new syntax. In contrast, our simulations suggest that this internalization may emerge from the *TC*, as the musical events with higher *TC* corresponded once again to the syntactically more important events. How does this finding relate to cross-cultural research on pitch structure? Indeed, the issues raised here are relevant for Western tonal music, but one can make the hypothesis that the same processes (perceptual, cognitive, memory, expectations) are likely to apply also to the processing of other musical systems (see, for example, Stevens, 2012).

Cross-cultural comparisons define a further interesting challenge of cognitive accounts. If participants highly familiarized with a given musical idiom (A) are presented with a new musical syntax (B), then their perceptual responses may be compared to those of another group of participants highly familiarized with this musical syntax B. Such a situation should presumably allow assessing the respective weight of acoustic and syntactic processes. Indeed, a syntactic approach would anticipate an assimilation of the new syntax to the mental schemata of the native idiom. As suggested previously (Meyer, 1956; Dowling, 1978; Curtis and Bharucha, 2009), Western listeners would assimilate nonwestern music to the scale and harmonic structures with which they were familiar. By contrast, the ASTM model would predict that responses of both groups of participants (Western and nonwestern) should be strongly influenced by the *TC* that prevails in each musical idiom. The ASTM model would thus anticipate more similarities between both groups than differences, these perceptual responses being all correlated with the changes in *TC* values in the ASTM memory. Moreover, the ASTM model cannot simulate acculturation processes as no long-term memory representation and learning processes are included.

Findings of Castellano et al. (1984) provided a strong support for the prediction of the ASTM model. A group of Western listeners and a group of Indian listeners were exposed to Indian musical excerpts that obey one of the rags common in North Indian music. After each musical excerpt, they were presented with each of the 12 tones of the chromatic scale, and evaluated how well each tone fitted musically with the rag context previously heard. The perceived hierarchy of tones fitted well with the Indian music hierarchy and the profiles for Western and Indian listeners were very similar: for individual rags, the intergroup correlations averaged 0.871 and were significant (*p* < 0.01) for each of the 10 rags. Moreover, it was stated that “[…] there is little evidence that the Western tonal hierarchies were dominant for either group of listeners.” (Castellano et al., 1984, p. 410). The authors explained this rather surprising finding by underlying that the “probe-tone ratings were consistently related to the distribution of tones in the contexts, which in general reflects scale membership.” (p. 411). Their analysis provided further evidence that the rating given to a tone was a function of the relative amount of time the tone sounded in the rag context (compared with other tones). These duration values were highly correlated with the rating profiles, *r* = 0.820, on average for the 10 rags, all were significant (*p* < 0.01), and this effect seemed as strong for Western as for Indians listeners (with *r* = 0.805 and 0.828, respectively). In order to interpret this strong effect of duration, the authors supposed that the Indian musical context presented before the rag (30 s of music, repeated two times) was too rich in terms of tones.

**Figure 16:**
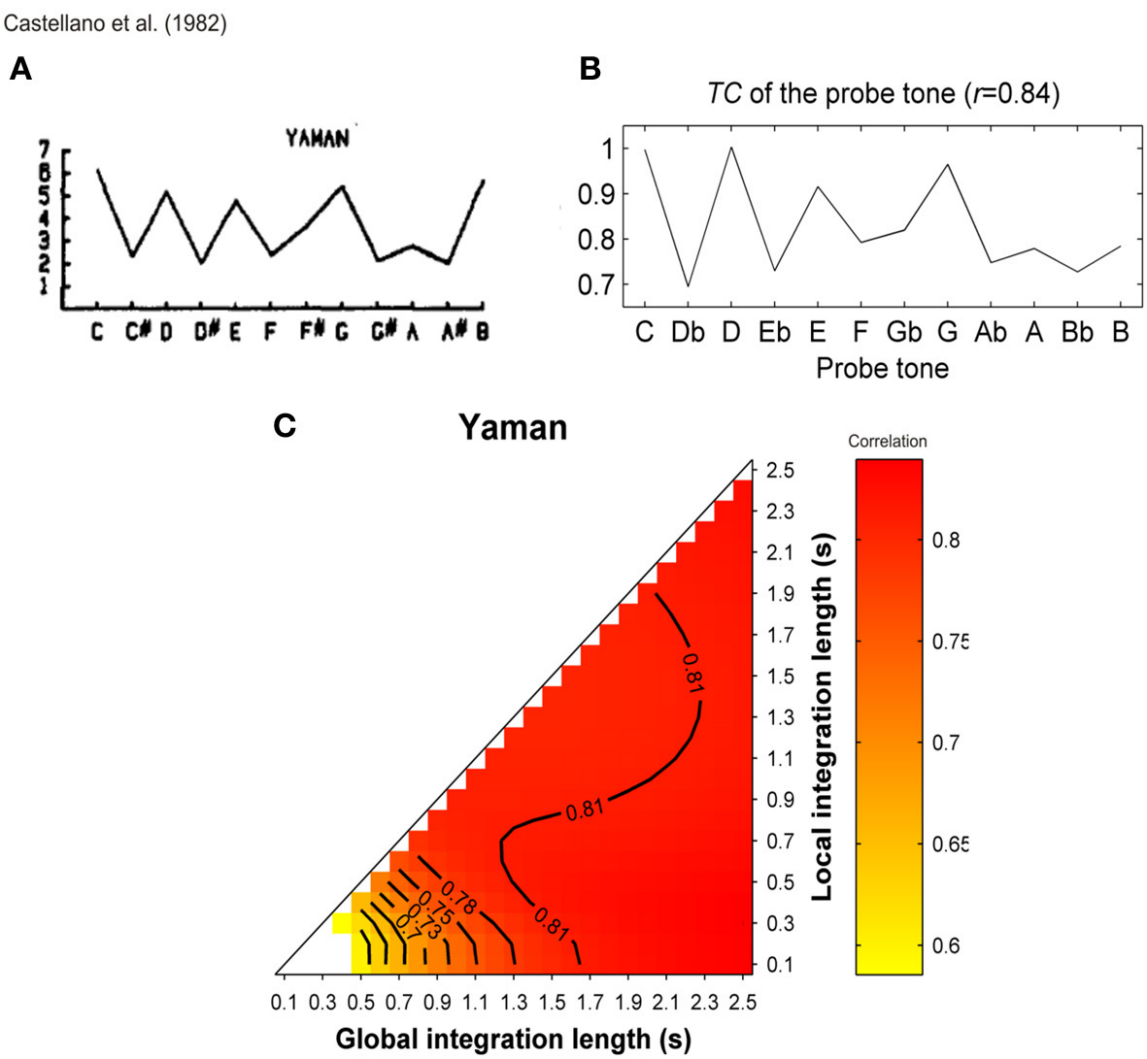
**Simulation of Castellano et al.'s (1984) study**. Panel **(A)** reports the ratings profile obtained with the *Yaman* rag, averaged over Western and Indian listeners. Panel **(B)** shows the simulated tone profile, computed with a local and global context integration windows of 0.3 and 2.5 s respectively. The correlation value between this simulated tone profile and the ratings equals 0.8396. Panel **(C)** displays the correlation values between the simulated tone profiles and the profiles obtained by Castellano et al. (1984) in the context of the *Yaman* rag, as a function of the local and global context integration windows. *r* coefficients are reported as contours.

These data are highly consistent with what may have been anticipated by the ASTM model, which is highly sensitive to the accumulation of acoustic information. Castellano et al. (1984) provided the complete description of the stimuli used in this study for only one rag (*Yaman*). For the purpose of the present simulation, the musical stimuli of Castellano et al. (1984) were generated, using the original spectral sound qualities, and the probe-tone test for this rag was administrated to the ASTM model. The model replicated the perceived tone hierarchies with a high level of accuracy: the tones with higher *TC* in this Indian rag correspond to the most hierarchical tones. The correlation value between *TC* values and both Indian and Western listeners were highly significant (ranking from *r* = 0.59 to 0.84, *p* < 0.05; see Figure 16)^5^. To further assess the explanation of the authors (i.e., the Indian melody used before the probe-tone test was too rich), we reran this simulation with only the short melody used before the presentation of each probe tone. The ASTM model nevertheless replicated the data of both groups, suggesting that using only this melody as test would have produced the same type of finding.

Concepts similar to echoic memory and “tonal contextuality” have appeared in previous works in which pitch and tonality perception are considered from the viewpoint of psychoacoustics and pattern processing (e.g., Parncutt, 1989; Leman, 1991a,b, 1992; Huron and Parncutt, 1993). This literature builds on ideas of von Helmholtz (1863/1912) and especially Terhardt et al. (1982a,b), in which perceptual features of pitch, chords, and tonality are predicted by a psychoacoustic model that integrates patterns over time, disregarding syntactic constructs.

Based on Hughes (1977), who did a tone duration analysis of one particular tonal composition, Schubert's Opus 94, No. 1, Krumhansl (1990) noted that the relative durations of the tones in this composition correlated almost perfectly with the probe-tone ratings (as measured by Krumhansl and Kessler, 1982) for the predominant key of the composition. Even the key-finding algorithms proposed in Krumhansl (1990) rest on ASTM: indeed, the algorithm evaluates the currently instilled key by combining the number of pitches and their duration (with or without decay of memory traces over time), which is in some way what the current model is doing too.

The fact that a similar effect of tone duration was reported here for Indian music, suggests that this effect is not specific to Western music. Moreover, a similar effect of tone duration was also reported for atonal music. In an atonal melody, when a tone is repeated, recognition memory for this tone is enhanced (Deutsch, 1972). All these findings suggest that perceived tone hierarchies are an emergent quality of ASTM. As quoted by Deutsch (1984), “[…], when listening to a piece, a hierarchy of memorability for the different notes in the piece is created. Certain notes become firmly embedded in memory; others, less firmly; and so on.” (p. 413). It is this hierarchy of memorability that is simulated by Leman's (2000) ASTM model, by taking into account not the symbolic representation of musical pitch, but all harmonic and subharmonic spectra inferred from the auditory signal by the neural encoding^6^.

(4) A key to understand the ASTM is that it does not just accumulate the harmonic (spectral) patterns that define the chord, but it also accumulates the subharmonic (auditory) patterns that are inferred from the tones and chords during auditory processing. These subharmonic patterns emerge from the autocorrelation analysis of the neuronal firing probabilities that reflect the frequencies in the stimulus (cf. the pitch periodicity analysis, Figure 1). Interestingly, the pitch images that come out of this model can also be conceived from the viewpoint of a geometrical representation. Several computer studies have shown that the geometry of the tonal space (as described by music theory, e.g., Krumhansl, 1979, 1990; Lerdahl, 2001) can be obtained by projecting these auditory images onto a two-dimensional map, using statistical principles of self-organization (Leman, 1991a,b, 1995; Blankertz et al., 1999; Janata et al., 2002; Toiviainen and Krumhansl, 2003). Given the fact that this projection is completely data-driven, it demonstrates that the geometry of the tonal space is inherent in the auditory images. Furthermore, the application of principal component analysis to these auditory images shows that this underlying geometry is low-dimensional (Leman, 1995), and therefore, that the tonal space can be captured in terms of a torus model, with chords and keys organized along the circle of fifths, as originally proposed by Krumhansl and Kessler (1982).

Listening to a chord progression leads to an integration of auditory images over a time-window, and the resulting integrated auditory image can be conceived as occupying a particular region of the tonal space, as if the tonal information contained in the music would highlight a certain spot on the torus model (see for example Janata et al., 2002, and the animations provided at http://atonal.ucdavis.edu/projects/musical_spaces). This spot or activation region thus represents a time-dependent context with which target chords can be compared. If the target chord falls outside this region, then the similarity will be low, if it falls inside this region, the similarity will be high (Leman, 1995). An important result of the computer simulations that resulted in these geometric models, is that the geometry is inherent in the acoustic signal of the context. Consequently, to account for context dependencies, it is not necessary to assume memory representations of an entire geometric model, nor top-town determinations of the target chords on the basis of a learned torus model or a related grammar. Instead, it seems sufficient to consider the emergence of signal-inherent local features, which amount to local regions in the geometrical space. As the simulations in the present paper show, this approach can predict (almost all) data. It demonstrates that certain psychological properties of music perception do not necessarily derive from the processing of rule-based systems or syntactic structures. Instead, these properties can be more parsimoniously explained by the perceptual contrasts that auditory-processed intrinsic physical features create with those of previous, contextual events accumulated in ASTM. The observation that some data cannot be simulated by the present ASTM suggests either shortcomings of this model (as discussed above) or the need to integrate the influence of some cognitive processes, linked to tonal knowledge stored in long-term memory. This later hypothesis requires the development of a model that considers both sensory and cognitive influences as a purely cognitive model cannot account for the data (e.g., the MUSACT model of Bharucha, 1987). Recently, Collins et al. (2014) proposed simulation work in this direction, notably by developing a model combined of sensory and cognitive representations to simulate tonal expectations in music.

## Conclusion

Understanding the neurocognitive foundation of musical syntax has numerous theoretical implications for cognitive sciences and evolutionary psychology (e.g., Pinker, 1997; Justus and Hutsler, 2005; Patel, 2010), and has several implications for clinical research (e.g., Patel, 2008). Up to now, it has been claimed that music is tapping into the neural resources of language for an integrative stage of processing that share functional similarity with language. However, the present finding leads to reevaluate the role played by auditory memory in syntactic-like processing. Despite some limitations of Leman's model, numerous data that were considered as demonstrating abstract computations in music were found to be parsimoniously accounted for by the accumulation of acoustic information in ASTM. This does not mean that musical syntax does not exist *per se*, or that some aspects of syntax processing could not tap into abstract computations. Our finding points to a considerable difference between music and language processing: low-level representation could be sufficient for numerous aspects of syntax processing (also including nonlocal dependencies) in music, while abstract levels of conceptual representation are indispensible for language processing. Therefore, the claim that music taps into the cognitive resources of language or uses the syntactic computational tool of language becomes a matter of debate. This debate should integrate (i) a full reevaluation of the power of auditory memory processes, and (ii) a critical evaluation of what musical syntax really means, especially also in relation to language.

### Conflict of interest statement

The authors declare that the research was conducted in the absence of any commercial or financial relationships that could be construed as a potential conflict of interest.

## Footnotes

^1^Several terms have been used to refer to hierarchical organization in music: musical stability/instability, relaxation/tension, harmonic relatedness, or distance, degrees of harmonic expectedness. In the present paper, we used the terms in quite equivalent meaning, following the terminology the authors of the quoted studies have used.

^2^This situation, however, leads to a positive bias for the cognitive interpretation, since every failure of the ASTM model can erroneously be interpreted as demonstrating the strength of the cognitive one.

^3^We are deeply grateful to A. D. Patel who has sent us the original MIDI sequences that were used to synthesize the stimuli with a piano timber.

^4^It would be premature to argue for a direct link between the electrophysiological responses and the model dynamics.

^5^Note, however, that although Western listeners behave like Indian listeners for the major part of the data, Indian listeners showed slightly higher ratings than Western listeners for one specific tone, i.e., the thât, a finding which cannot be accounted for by the present ASTM model and thus points to tonal acculturation with the Indian system.

^6^Our interpretation based on memorability is also consistent with the weak differences between musicians and nonmusicians in musical syntax perception reported by different research teams (see Bharucha and Stoeckig, 1987; Koelsch et al., 2000; Justus and Bharucha, 2001; Bigand and Poulin-Charronnat, 2006).
